# Comprehensive analysis of Fc-mediated IgM binding to the *Plasmodium falciparum* erythrocyte membrane protein 1 family in three parasite clones

**DOI:** 10.1038/s41598-019-42585-0

**Published:** 2019-04-15

**Authors:** Maria del Pilar Quintana, Gertrude Ecklu-Mensah, Sergey O. Tcherniuk, Sisse Bolm Ditlev, Andrew V. Oleinikov, Lars Hviid, Mary Lopez-Perez

**Affiliations:** 1 Centre for Medical Parasitology at Department of Immunology and Microbiology, Faculty of Health and Medical Sciences, University of Copenhagen, Copenhagen, Denmark; 2 Department of Infectious Diseases, Rigshospitalet, Copenhagen, Denmark; 3 Department of Immunology, Noguchi Memorial Institute for Medical Research, University of Ghana, Legon, Ghana; 4 Charles E Schmidt College of Medicine, Florida Atlantic University, Boca Raton, FL USA

## Abstract

PfEMP1 is a family of adhesive proteins expressed on the surface of *Plasmodium falciparum*-infected erythrocytes (IEs), where they mediate adhesion of IEs to a range of host receptors. Efficient PfEMP1-dependent IE sequestration often depends on soluble serum proteins, including IgM. Here, we report a comprehensive investigation of which of the about 60 *var* gene-encoded PfEMP1 variants per parasite genome can bind IgM via the Fc part of the antibody molecule, and which of the constituent domains of those PfEMP1 are involved. We erased the epigenetic memory of *var* gene expression in three distinct *P. falciparum* clones, 3D7, HB3, and IT4/FCR3 by promoter titration, and then isolated individual IEs binding IgM from malaria-unexposed individuals by fluorescence-activated single-cell sorting. The *var* gene transcription profiles of sub-clones measured by real-time qPCR were used to identify potential IgM-binding PfEMP1 variants. Recombinant DBL and CIDR domains corresponding to those variants were tested by ELISA and protein arrays to confirm their IgM-binding capacity. Selected DBL domains were used to raise specific rat anti-sera to select IEs with uniform expression of candidate PfEMP1 proteins. Our data document that IgM-binding PfEMP1 proteins are common in each of the three clones studied, and that the binding epitopes are mainly found in DBLε and DBLζ domains near the C-terminus.

## Introduction

Malaria is caused by protozoan parasites of the genus *Plasmodium*. There are an estimated 219 million malaria cases annually, including about 435,000 fatal episodes, mainly caused by infection with *P. falciparum*^1^. The particular virulence of this species is related to its multiple strategies to evade acquired host immunity. Important among them is the capacity of *P. falciparum*-infected erythrocytes (IEs) to sequester in various tissues to avoid immune clearance in the spleen^2^. The *P. falciparum* erythrocyte membrane protein 1 (PfEMP1) family is essential for the adhesion of IEs to host cell membrane receptors (on endothelial cells or erythrocytes), which in some cases requires soluble host proteins^3–7^.

The acquisition of PfEMP1-specific protective immunity to prevent IE sequestration is frustrated by the parasite’s ability to express a single PfEMP1 variant at the IE surface at a time, and to switch among the ~60 PfEMP1 proteins encoded by the *var* gene family^8^. The *var* genes can be divided into several groups based on genomic location, direction of transcription, and structural features^9,10^. Expression of PfEMP1 variants encoded by particular *var* gene groups (and their sub-groups) has been associated with discrete clinical presentations and IE adhesion to specific host receptors. Thus, PfEMP1 expression in parasites isolated from severe malaria patients with little or no pre-acquired immunity is often dominated by variants encoded by the relatively conserved Group A genes. The more diverse Group B and Group C genes are more commonly found among uncomplicated and asymptomatic infections, while the Group E genes that encode VAR2CSA-type PfEMP1 variants are responsible for the pathogenesis of placental malaria^11^. These different subsets of PfEMP1 proteins contain specific Duffy binding-like (DBL) domains and cysteine-rich inter-domain regions (CIDR). Both DBL and CIDR domains can be divided into structurally related classes (α, β, γ, δ, ε, ζ, and α, β, γ, respectively)^12^. DBL and CIDR domains mediate IE adhesion to various host receptors, such as endothelial protein C receptor (EPCR), intercellular adhesion molecule 1 (ICAM-1), CD36, and oncofetal chondroitin sulfate^13–18^.

A handful of PfEMP1 variants has been reported to bind IgM via the Fcµ region of the antibody rather than by the hypervariable, antigen-specific Fab fragment^19^ (we will refer to this type of IgM-binding as “non-immune”, as it is specific in the sense that it depends on Fcµ but is independent of the antigen-specificity of the IgM molecules involved). We recently reported the existence of four additional non-immune IgM-binding PfEMP1 variants in *P. falciparum* 3D7 sub-clones. Relatively few sub-clones and PfEMP1 variants were tested, and the study thus probably underestimated the total number of non-immune IgM binders^5^. Furthermore, the study did not assess potential inter-clonal variation in the capacity for Fc-mediated binding of IgM to PfEMP1. To overcome these limitations, we report here results from single-cell sorting of parasite populations with highly heterogeneous *var* gene transcription to obtain a comprehensive mapping of non-immune IgM-binding PfEMP1 variants in the three distinct *P. falciparum* clones 3D7, HB3, and IT4/FCR3 (subsequently referred to as IT4). We also probed a multiplex array of PfEMP1 domains from *P. falciparum* 3D7 with non-immune IgM. Together with recombinant PfEMP1 proteins and specific rat anti-sera the study allowed us to identify several new PfEMP1 variants and constituent domains involved in non-immune IgM binding to IEs.

## Results

### Erasure of epigenetic memory by pVBH transfection

At the outset, our *P. falciparum* clones 3D7, HB3, and IT4 dominantly transcribed one *var* gene each (*pfd1005c*, *hb3var06*, and *it4var60*, respectively) (Fig. S1, left panels). To obtain *P. falciparum* populations with as heterogeneous *var* gene transcription as possible, we transfected each of the three *P. falciparum* clones with the pVBH plasmid, which contains a blasticidin S deaminase (*bsd*) gene under the control of a *var* promoter^20,21^. For each of the clones, selection by blasticidin pressure for high copy numbers of this plasmid markedly reduced transcription of the endogenous *var* genes (Fig. S1, center panels). Two weeks after release from the drug pressure, *bsd* transcription had decreased and the parasites transcribed a diverse set of endogenous *var* genes, without dominance of the initially most abundant *var* gene transcript (Fig. S1, right panels). This indicates efficient erasure of epigenetic memory, as previously described^20,21^.

### Identification of candidate IgM-binding PfEMP1 proteins by single-cell sorting of infected erythrocytes

**Figure 1:**
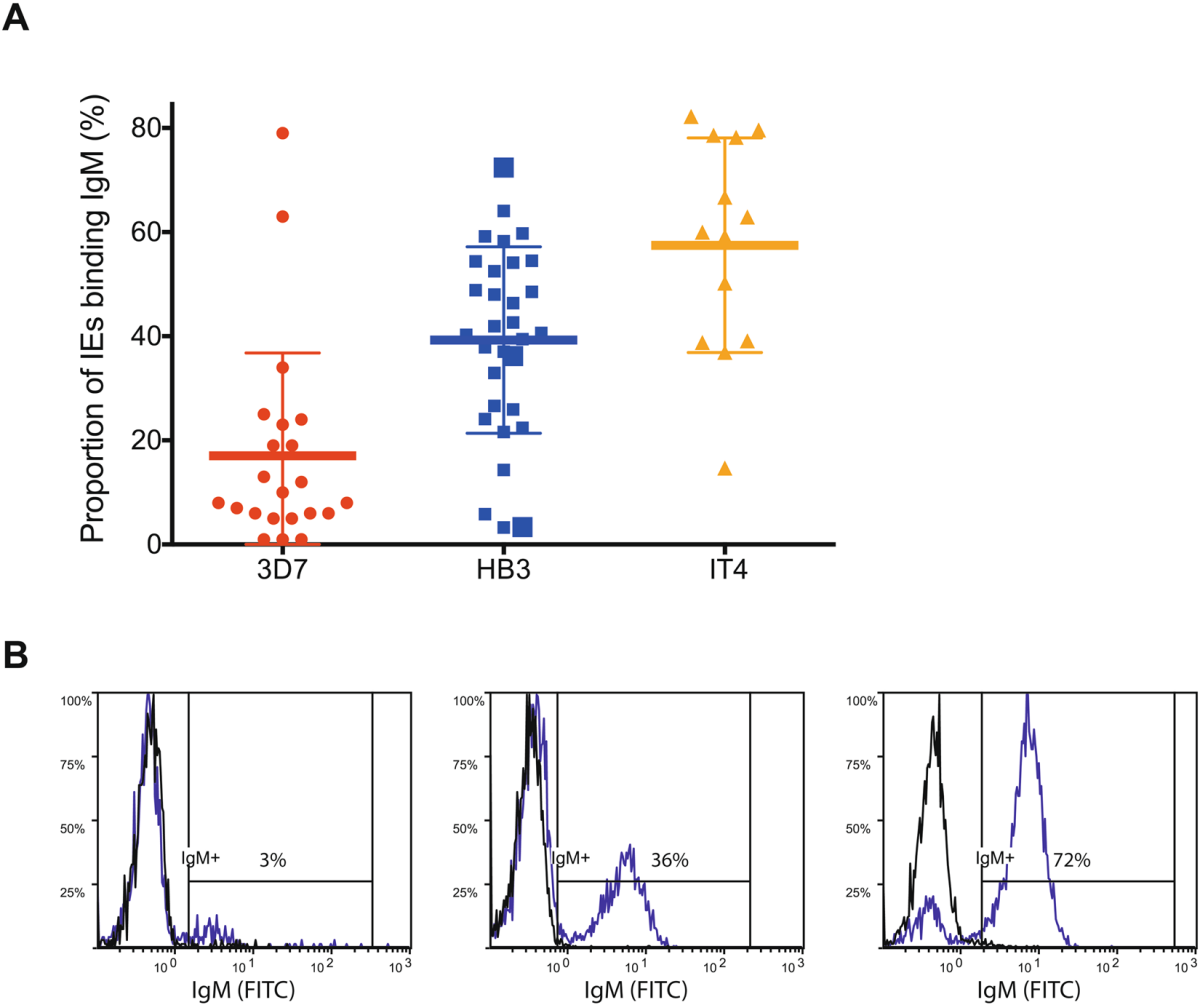
Non-immune IgM Binding to *P. falciparum*-infected erythrocytes. Percentages of IgM-binding late-stage IEs in individual IgM^+^ sub-clones of 3D7 (red circles, n = 22), HB3 (blue squares, n = 31), and IT4 (orange triangles, n = 13). (**A**) Means and standard deviations are shown. Representative flow cytometry histograms of three HB3 sub-clones with high, medium, and low percentages of IgM^+^ IEs (identified in A by larger symbols) in the presence (blue) and absence of non-immune IgM (black) (**B**).

**Figure 2:**
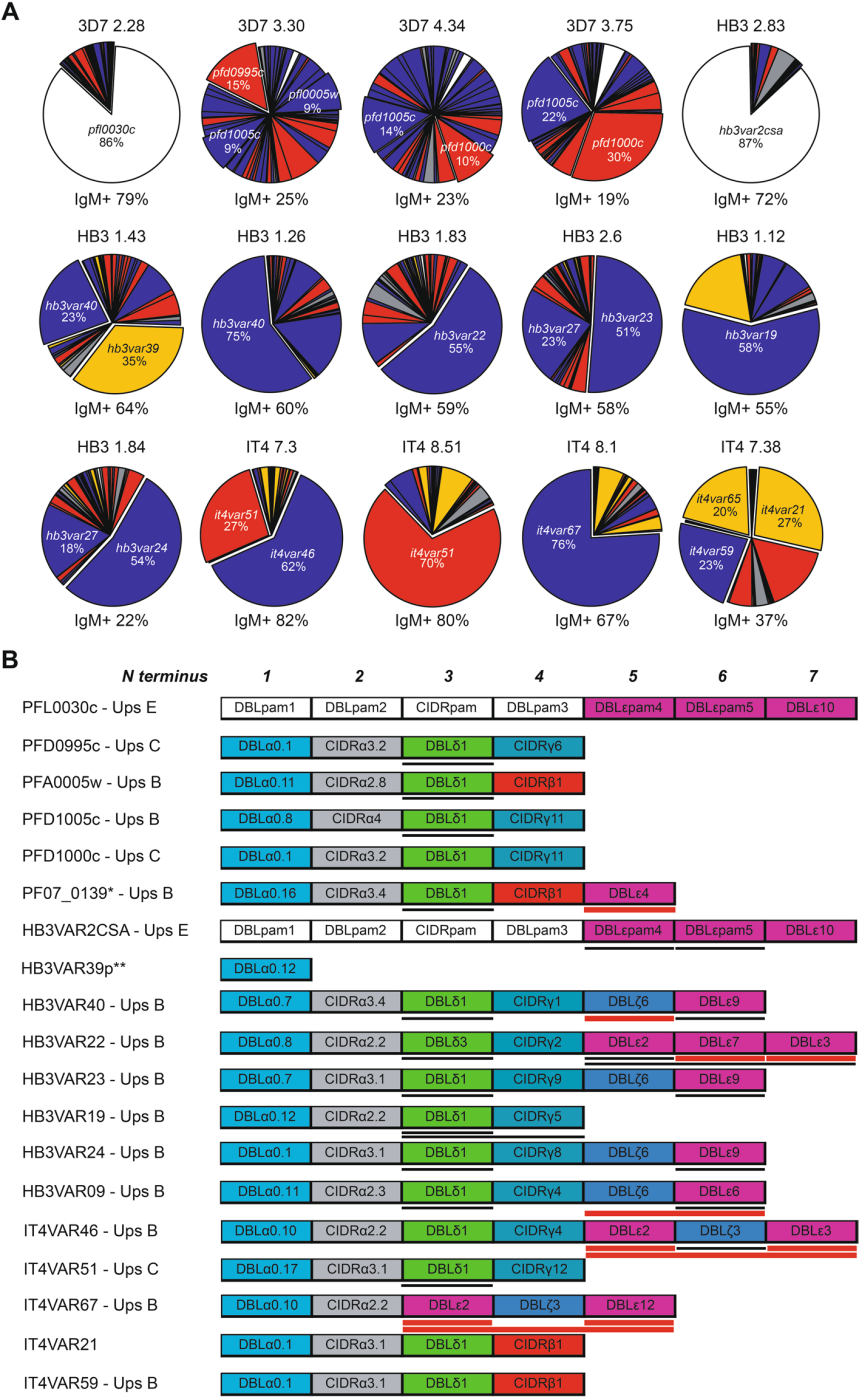
Var gene transcription in *P. falciparum* IgM-selected sub-clones. Relative proportions of *var* gene transcripts in a selected subset of IgM-binding sub-clones of 3D7, HB3, and IT4 (additional data in Fig. S2 and Tables S1–S3). (**A**) Transcripts are color coded according to *var* Group (A: gray; B: blue, C: red, E: white, uncharacterized: yellow)^5^. Major transcripts are identified by name, and all candidate genes selected are shown as “exploded pie slices”. The percentage of corresponding IEs binding IgM is indicated below each pie diagram. Domain structures of the PfEMP1 proteins encoded by the named major transcripts in (A), color-codes as before^5^. (**B**) The orientation and domain numbers are indicated in italics along the top of the panel. Recombinant PfEMP1 constructs used in the present study are indicated by underlining, with those confirmed by ELISA to bind non-immune IgM identified by heavy red lines. *Identified as a candidate IgM-binder by Jeppesen *et al*.^5^. **Only the sequence of the DBLα_D1 domain of the PfEMP1 is known (http://www.cbs.dtu.dk/services/VarDom/).

Erythrocytes infected with late-stage parasites, in which the *var* gene epigenetic memory had been erased by *bsd* transfection and drug selection, were labeled with non-immune IgM and subjected to fluorescence-activated single-cell sorting to isolate individual IgM^+^ IEs. After *in vitro* expansion of the sorted IEs for 3–6 weeks, we obtained 66 IgM-reactive sub-clones with varying proportions of IgM^+^ IEs (Fig. 1). Thirty-four sub-clones transcribed a dominant (≥35%) *var* gene (Figs 2, S2, Tables S1–S3). The relative level of dominant *var* gene transcripts generally reflected the IE reactivity with non-immune IgM. This correspondence, and analysis of the most dominant transcripts, were used to identify genes likely to encode IgM-binding PfEMP1 antigens in the three parasite clones. Apart from the Group E genes encoding VAR2CSA-type PfEMP1 in the 3D7, HB3, and IT4 (*pfl0030c*, *hb3var2csaA* and *hb3var2csaB*, and *it4var04*, respectively), all the identified genes belong to Group B or Group C. Many did not encode any DBLε and DBLζ domains, which have previously been associated with non-immune IgM binding to PfEMP1^4–6,22–24^ (Figs 2, S2). Instead, they encoded a DBLδ1 domain near the C-terminus, which thus appeared to represent a hitherto unidentified IgM-binding domain type (Figs 2, S2). A few of the sub-clones showed low (≤15%) IgM^+^ IEs despite transcribing a single dominant *var* gene (Tables S1–S3), probably because they had largely switched away from the *var* gene encoding the IgM-binding PfEMP1 detected at the time of the single-cell sorting. Alternatively, these sub-clones were false positives not expressing an IgM-binding PfEMP1 at the time of sorting. Regardless of the reason for the IgM non-reactivity, this information was taken as an indication of unlikely candidates for genes encoding IgM-binding PfEMP1 variants. Examples include PFL13_0001, PFL2665c, and PF08_0107 (Table S1), HB3VAR27, HB3VAR28, HB3VAR29 (Table S2), and IT4VAR15 (Table S3).

### Identification of non-immune IgM-binding domains in candidate recombinant PfEMP1 proteins

**Figure 3:**
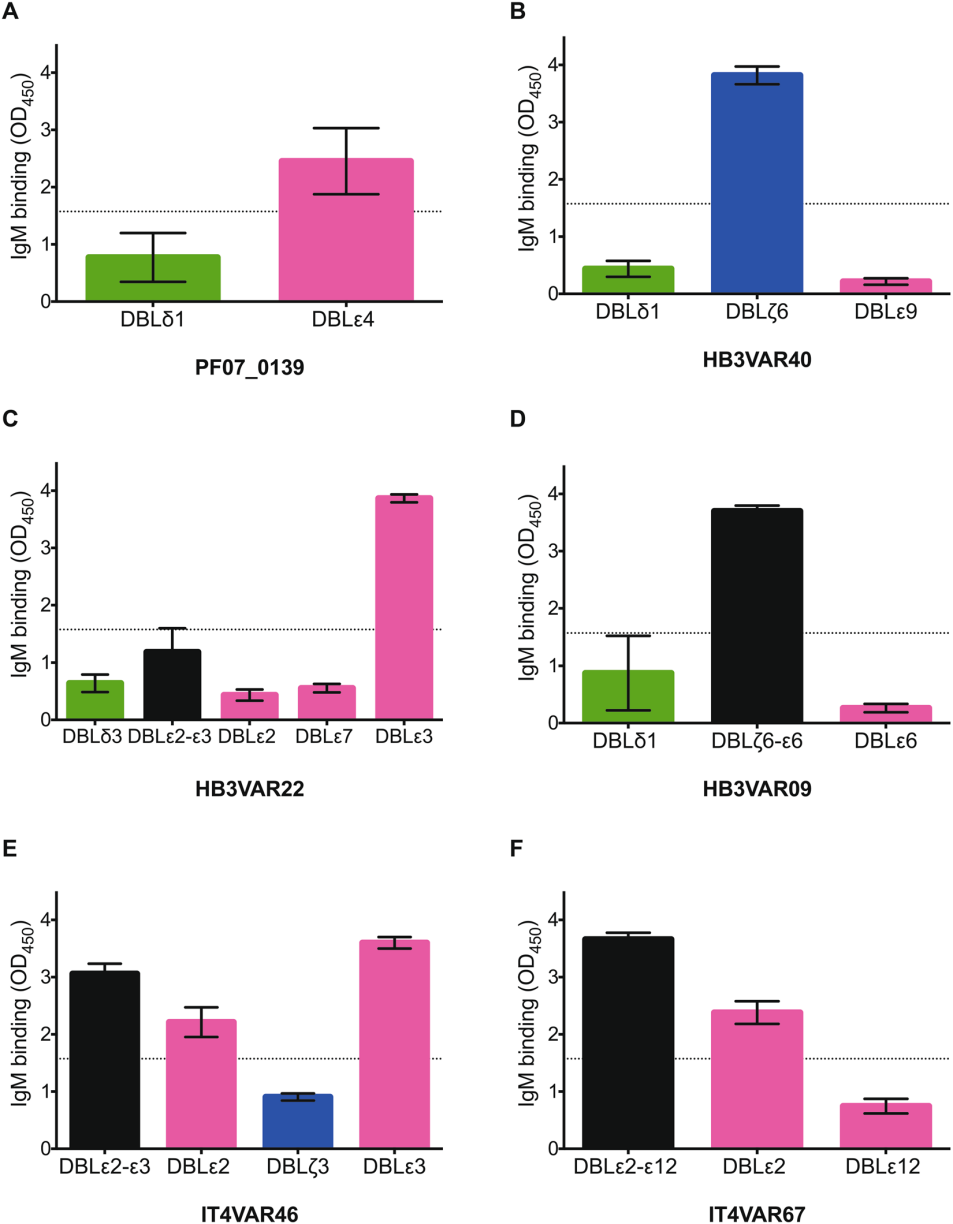
Non-immune IgM-binding to recombinant PfEMP1 domains in ELISA. Binding of non-immune IgM to immobilized recombinant proteins representing candidate IgM-binding domains in PF07_0139 (**A**), HB3VAR40 (**B**), HB3VAR22 (**C**), HB3VAR09 (**D**), IT4VAR46 (**E**), and IT4VAR67 (**F**). Means and standard deviations of data from three independent experiments are shown. Dotted lines represent the cut-off above which binding to non-immune IgM was considered as positive. Domain types are color-coded as in Fig. 2B.

To examine further the IgM-binding properties of the candidate PfEMP1 proteins, we generated 38 recombinant domains belonging to 20 different PfEMP1 variants in 3D7 (n = 7), HB3 (n = 10), and IT4 (n = 3) (Figs 2, S2). Domains with substantial IgM-binding capacity were identified by ELISA near the C-terminus of PF07_0139 (DBLε4), HB3VAR40 (DBLζ6), HB3VAR22 (DBLε3), HB3VAR09 (DBLζ6- DBLε6), IT4VAR46 (DBLε2 and DBLε3), and IT4VAR67 (DBLε2) (Fig. 3), whereas the DBLδ1-type or DBLε9-type candidate domains generally showed limited affinity for non-immune IgM in ELISA (Figs 3 and S3). Results obtained with a “reversed” ELISA, where the plates were coated with IgM rather than the recombinant proteins, generally yielded similar results, except for HB3VAR22 and IT4VAR67 where binding to IgM was detected for additional domains (DBLε7 and DBLε12, respectively) (Fig. S4).

In a complementary approach, we tested a BioPlex bead array composed of 157 recombinant proteins representing almost all DBL and CIDR domains in the PfEMP1 proteins of *P. falciparum* 3D7 for binding by non-immune IgM, in a manner similarly to that used previously in tests of PfEMP1 binding to the endothelial receptors ICAM-1, CD36, and αVβ3 and αVβ6 integrins^25–27^. By this method, evidence of non-immune IgM binding was found in constructs from 13 PfEMP1 variants in *P. falciparum* 3D7 (Fig. S5). The data corroborated the IgM-binding capacity of domains in MAL6P1.4 (DBLε3), PFL0020w (DBLε4), and PF07_0139 (DBLε4) detected by cell-sorting and ELISA previously^5^ or here (Fig. S5A). With this approach, we also obtained preliminary evidence of additional IgM-binding domains in MAL6P1.4 (DBLγ13) and PFL0020w (DBLβ5), and in the head structure of MAL6P1.316 (DBLα2-CIDRα1.8) (Fig. S5A,C). MAL6P1.316 has previously been implicated in non-immune IgM binding, but the domain involved was not identified^5^. Furthermore, DBLα2 has been implicated in rosetting^28^, and CIDRα1.8 in IE adhesion to EPCR^29^. The BioPlex array also pointed to several PfEMP1 variants and domains not previously associated with non-immune IgM-binding. These included C-terminal domains in PF11_0008 (DBLδ5-CIDRβ4 and DBLβ9), PF08_0141 (DBLζ5), PF08_0103 (DBLδ1-CIDRβ1), and PFC0005w (DBLδ1-CIDRβ1) (Fig. S5B,C). Expression of PF08_0103 was recently associated with sporozoite-stage *P. falciparum* parasites, and PF08_0103-specific antisera blocked sporozoite invasion of hepatocytes^30^. However, it is unknown if these and other observations^31,32^ on these particular PfEMP1 variants are of any significance to the present findings. Finally, the array revealed non-immune IgM-binding in the N-terminal head structures of several additional PfEMP1 variants (PFE0005w, PFD0020c, PF11_0007, PF07_0048, and PF07_0051 (Fig. S5B). A weak IgM response to PF07_0051-DBLα1 was previously noted in an experimentally infected volunteer^33^. Overall, the BioPlex findings suggest that the capacity for Fc-mediated IgM-binding may be even more widespread than the single-IE sorting and PfEMP1-specific antibody selection experiments suggest. As such, they provide leads for future studies, ideally employing IEs expressing the corresponding native proteins.

### Verification of the capacity of native candidate PfEMP1 proteins to bind non-immune IgM

**Figure 4:**
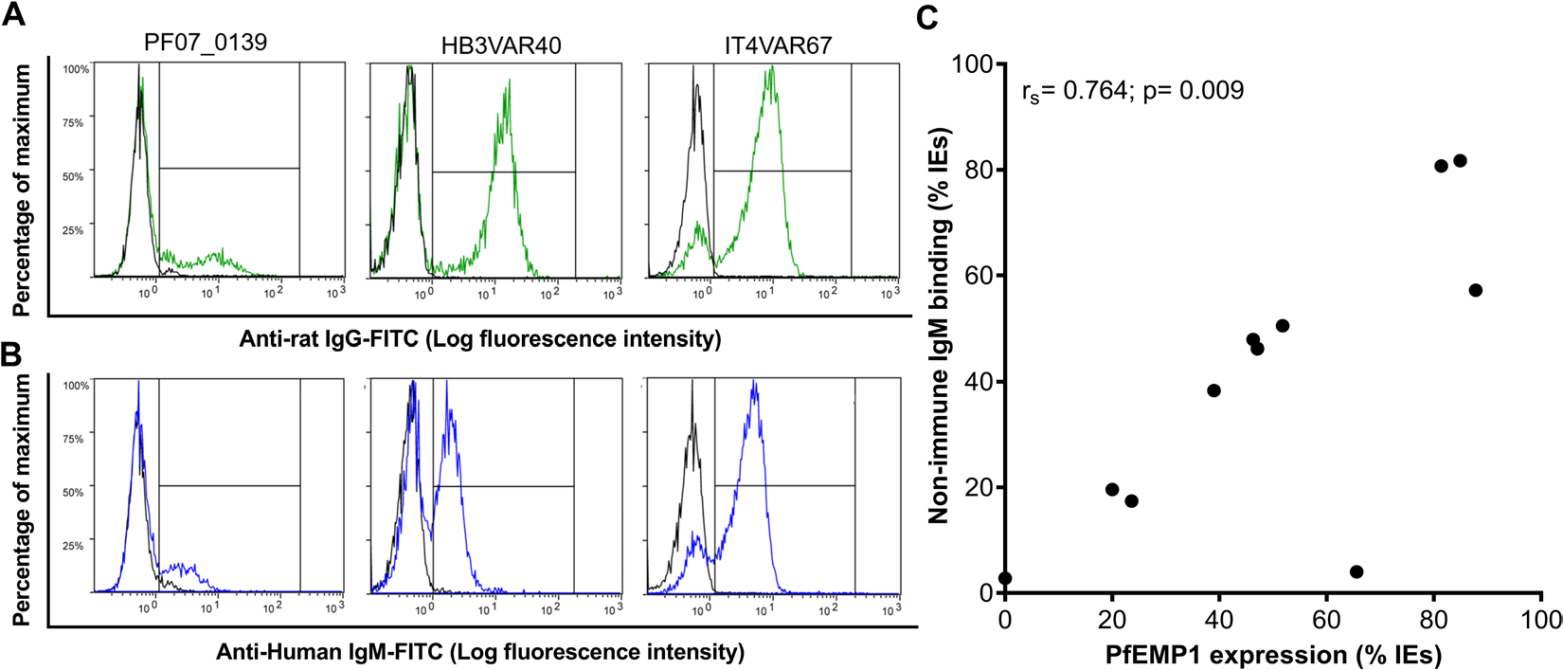
Non-immune IgM-binding and specific PfEMP1 expression on the surface of erythrocytes infected by *P. falciparum* sub-clones. Representative sub-clones generated by single-cell sorting and labeled with rat IgG specific for the PfEMP1 corresponding to the dominantly transcribed *var* gene (green) (**A**), or non-immune IgM (**B**). Background labeling indicated in black. Correlation (r_s_) of non-immune IgM-binding and labeling by PfEMP1-specific IgG for all sub-clones (**C**).

**Figure 5:**
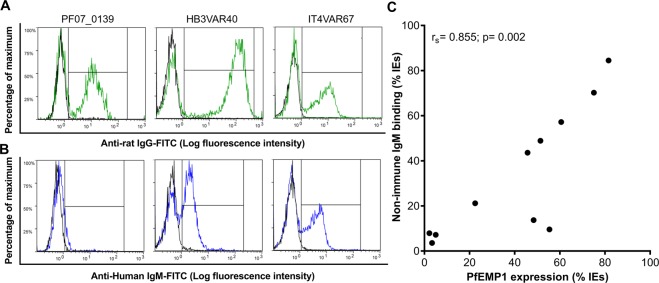
Non-immune IgM-binding and specific PfEMP1 expression on the surface of infected erythrocytes selected by rat anti-PfEMP1 domain-specific IgG. *P. falciparum* 3D7 (left), HB3 (center), and IT4 (right) with heterogeneous *var* gene transcription (Fig. S1) after several rounds of selection for IE reactivity with rat anti-PF07_0139, anti-HB3VAR40, and anti-IT4VAR67, respectively, and labeled with the antibody used for selection (**A**) or non-immune IgM (**B**). Background labeling indicated in black. Correlation (r_s_) of non-immune IgM-binding and labeling by PfEMP1-specific IgG for all selected parasites expressed as percentage of IEs (**C**).

To further strengthen the link between IE surface-expression of particular native PfEMP1 variants and non-immune IgM binding to the IEs, we selected 12 recombinant domains (from 11 PfEMP1 variants) binding or not binding non-immune IgM in ELISA (Table S4). The selected domains were used to generate specific rat anti-sera and purified domain-specific IgG. In most cases, labeling of IEs from the single cell-sorted sub-clones with the domain-specific IgG (Figs 4A; S6A) and non-immune IgM (Figs 4B; S6B) demonstrated strong positive correlation between the percentage of IEs expressing candidate non-immune IgM-binding native PfEMP1 variants and the percentage of IEs that indeed bound non-immune IgM (Fig. 4C). The outlier (Fig. 4C) is HB3VAR19, where we could not verify IgM-binding to the candidate domains by ELISA (Fig. S3). The ability of this PfEMP1 protein to bind non-immune IgM must thus requires further investigation. Furthermore, repeated selection of pVBH-transfected parasites (after two weeks of culture without blasticidin pressure) with domain-specific rat IgG generally led to increased IE surface expression of the cognate native PfEMP1 variant (Figs 5A; S7A) and of non-immune IgM binding (Figs 5B; S7B), with a strong positive correlation between the two variables (Fig. 5C). The outliers (Fig. 5C) are HB3VAR19 (see above) and PF07_0139, which was found to bind IgM by ELISA (Fig. 3A) and Bioplex (Fig. S5A). However, we previously reported that the IgM-binding to native PF07_0139 on IEs surface is very low^5^, and the present data support that conclusion. Taken together, the data in sub-clones and selected parasites support the capacity of native PF07_0139, HB3VAR09, HB3VAR22, HB3VAR23, HB3VAR24, HB3VAR40, IT4VAR46, IT4VAR51, and IT4VAR67, to bind non-immune IgM.

Overall, our data strongly support that binding of non-immune IgM to the surface of IEs is common (probably > 10% of all PfEMP1), mediated by particular PfEMP1 variants, and can be found in genetically diverse *P. falciparum* clones.

## Discussion

*P. falciparum* causes the most severe form of malaria in humans. This is not least due to the evolution in this parasite species of multiple ways to evade host innate and acquired immune responses^34^. These include IE misappropriation of host plasma proteins^7,35,36^, endothelial sequestration of IEs^37,38^, and clonal antigenic variation^39,40^. The PfEMP1 family of adhesive proteins, expressed on the IE surface, are involved in all these processes^4,41,42^. In previous work, others and we have shown that Fcμ-mediated (i.e., non-immune) binding of IgM to particular PfEMP1 variants can function to shield IEs from recognition by PfEMP1-specific IgG^43^, and may also serve to augment IE adhesion to endothelial receptors^6,44^. Whatever function PfEMP1-bound IgM serves, it appears to be of clinical significance. The phenotype has repeatedly been associated with severe malaria in children, caused by parasites expressing Group A PfEMP1 variants^3,6,28^ and with malaria in pregnant women, caused by parasites expressing Group E (VAR2CSA-type) PfEMP1^24,43,45,46^. Furthermore, it has been proposed that IgM-binding to several Group B PfEMP1 variants^5^, which are otherwise generally associated with uncomplicated malaria and asymptomatic infections, serves to increase the adhesive strength between IEs and endothelial receptors^6,7,44^. This idea supports the importance of the non-immune IgM-binding phenotype.

Both DBL and CIDR domains in the N-terminal head structure^22,46^ and C-terminal DBL domains^4–6,46,47^ have previously been implicated as the domains mediating non-immune binding of IgM to PfEMP1. In the single previous study assessing how many of the about 60 PfEMP1 variants encoded by a specific *P. falciparum* genome have the capacity to bind IgM that way, five were identified using the 3D7 clone^5^. In four of those, IgM bound to a C-terminal ε- or ζ-type DBL domain, whereas the PfEMP1 domain involved in the low-intensity IgM binding to PF07_0139 was not identified^5^. However, only a limited number of IgM-binding sub-clones were studied and the involvement of N-terminal domains was not systematically assessed. It is thus likely that additional IgM-binding PfEMP1 variants exist. Moreover, the study included only a single parasite clone.

To overcome these limitations, the current study was undertaken to provide an exhaustive analysis of non-immune IgM-binding PfEMP1 variants in the three *P. falciparum* clones 3D7^48^, HB3^49^, and IT4^40,50^. The three parasite clones are representative of isolates from Africa, Central America, and Asia, respectively^51^. Furthermore, their *var* gene repertoires are highly divergent, with only two conserved genes (*var1csa* and *var2csa*) in common^52^.

We used two complementary approaches to identify PfEMP1 variants capable of binding non-immune IgM via Fcµ and to pinpoint the constituent domains involved in candidate proteins. In one approach, we used fluorescence-activated single-cell sorting to sub-clone individual IgM^+^ IEs from each of the three parasite clones (Fig. 1), followed by analysis of *var* gene transcription profiles and production of recombinant proteins representing specific domains in candidate PfEMP1 variants^5^ (Figs 2 and S2). The recombinant proteins were subsequently used to test their IgM-binding capacity directly (Figs 3 and S3). We also used them to generate antisera for detection of PfEMP1 expression on IgM-binding sub-clones (Figs 4 and S6). Finally, we used the PfEMP1-specific rat IgG to select erythrocytes infected by parasites with diverse *var* gene transcription (Fig. S1) for IE surface expression of the corresponding native protein and test their IgM-binding capacity (Figs 5 and S7). In the other approach, we made use of a protein array containing constructs representing almost all PfEMP1 domains or domain tandems (DBL-CIDR) in *P. falciparum* 3D7, immobilized on BioPlex beads^26,27^ (Fig. S5). Using these two approaches, we confirm the IgM-binding capacity of the PfEMP1 proteins and C-terminal DBLε and DBLζ domains implicated in our previous studies^5,6^. Several candidate IgM-binding PfEMP1 proteins also contained C-terminal DBLδ domains, but their capacity to bind IgM was lower (Figs 3, S3, S4). In addition, we document for the first time the IgM-binding capacity of Group E PfEMP1 in HB3 (HB3VAR2CSA) (Table S2) and of several C-terminal DBLε and DBLζ domains (PF07_0139, HB3VAR09, HB3VAR22, HB3VAR40, IT4VAR46, and IT4VAR67) (Table S1–S3). Finally, we provide preliminary evidence of IgM-binding DBLβ and DBLγ domains nearer the N-terminus (MAL6P1.4, PFL0020w) and C-terminus (PF11_0008), as well as fragments containing DBLα-CIDRα and DBLδ-CIDRβ domains in a number of PfEMP1 variants (Fig. S5). Most of those domain classes have not previously been implicated in Fcµ-mediated binding of IgM.

In other PfEMP1 variants implicated by sub-cloning of IgM^+^ IEs, it was not possible for us to confirm the IgM-binding capacity of selected candidate domains by ELISA (e.g., HB3VAR23_DBLε and HB3VAR24_DBLε) (Fig. S3). However, specific antisera against those domains could be used to select IEs that clearly bound non-immune IgM (Fig. S7), suggesting that the IgM-binding domains in those variants are different from those selected as candidates by us.

Overall, our study provides the first comprehensive overview of PfEMP1 proteins and domain classes that can bind IgM via its Fcµ domain. We document that the capacity for PfEMP1-dependent binding of IgM to the IE surface is prevalent in several distinct *P. falciparum* genomes, and show that some PfEMP1 variants (e.g., MAL6P1.4, HB3VAR22, IT4VAR46, and IT4VAR67) appear to possess several IgM-binding domains. Finally, our investigation indicates that non-immune IgM-binding may also occur among DBLα-CIDRα, DBLδ1, DBLβ, and DBLγ-type domains. However their ability to bind IgM appears less pronounced than that of DBLε- and DBLζ-type domains, and the biological relevance of this part of the study should be assessed further. While we did not investigate the functional significance of non-immune IgM-binding in the present study, previous reports have indicated roles in immune-evasion as well as pathogenesis (enabling rosetting and enhancing endothelial adhesion)^6,43,44^. Together, these findings support the idea that non-immune IgM binding confers survival advantages on parasites expressing PfEMP1 variants with IgM-binding domains, thereby rendering this phenotype of likely clinical importance.

## Methods

### Parasite *in vitro* culture and transfection

The *P. falciparum* clones 3D7, HB3, and IT4 were maintained *in vitro* in blood group O erythrocytes and serum-free medium, essentially as described before^53^. The 3D7 clone, transfected with the plasmid pVBH to erase epigenetic memory and achieve heterogeneous *var* gene transcription^54^, was generated and kindly provided by Ron Dzikowski^21^. Corresponding HB3 and IT4 parasites were generated by us in a similar way, except that the blasticidin concentration was increased to 10 µg/mL to recover parasites carrying high plasmid copy numbers. The genotypic identity of the parasites and the absence of *Mycoplasma* infection in the cultures were regularly verified.

### Fluorescence-activated single-cell sorting of IgM-positive infected erythrocytes

Erythrocytes infected with pVBH-transfected late-stage parasites, obtained 14 days after release from blasticidin drug pressure, were purified by magnetic-activated cell sorting (MACS) and labeled with non-immune human IgM (10 nM; Sigma). After thorough washes in sterile phosphate-buffered saline supplemented with fetal bovine serum (2%), IEs were stained with phycoerythrin-conjugated anti-human IgM (1:200; Jackson ImmunoResearch) and washed as above. A BD FACSJazz cell sorter (BD Biosciences) was used to sort individual IgM-binding IEs into round-bottom 96-well plates (Thermo Fisher Scientific) containing serum-free culture medium (100 µL) and uninfected erythrocytes (1 µL). The parasites were maintained in the plates for 2-4 weeks as described above, changing medium twice weekly, alternating between medium alone and medium with erythrocytes as above. Parasite growth was monitored regularly by microscopy, testing small aliquots from 10 random wells per plate. Wells with microscopic evidence of parasite multiplication were transferred to culture flasks and maintained until stably growing parasite sub-clones were established.

### *var* gene transcription profiling

Total RNA was prepared from synchronous ring-stage IEs using TRIzol (Ambion). Genomic DNA was removed by DNase I treatment (Invitrogen) and cDNA was generated using SuperScript II reverse transcriptase and random primers (Invitrogen) according to the manufacturer’s instructions. The cDNA, QuantiTec SYBR Green PCR Master Mix (Qiagen), and three validated sets of *var* gene-specific primers were used to assess *var* gene transcription profiles by real-time quantitative PCR on a Rotorgene RG-3000 thermal cycler (Corbett Research), as described in detail elsewhere^55–57^. The transcription levels for each *var* gene was calculated using the 2^−ΔCT^ method, relative to the levels of the housekeeping gene seryl-tRNA synthetase.

### Recombinant protein expression

Recombinant proteins with N-terminal his-tags and representing various PfEMP1 domains were produced in *Escherichia coli* Shuffle C3030 cells (New England Biolabs). Constructs were amplified from parasite genomic DNA, using specific primer sets (Table S5) and cloned into the pET15b modified plasmid^58^. All expressed proteins were purified by immobilized metal ion affinity chromatography using His-Trap High Performance columns (GE Healthcare). The purity of each protein was analyzed by sodium-dodecyl-sulfate polyacrylamide gel electrophoresis, followed by Coomassie staining with InstantBlue (Expedeon) following the manufacturer’s instructions. His-tags were detected by western blot using HRP-coupled anti-his antibodies (Qiagen) (Fig. S8).

### Production of PfEMP1-specific anti-sera

Three male Wistar rats per domain were immunized by subcutaneous injection four times at two-weeks intervals with 30 μg (first injection) and 15 µg (subsequent injections) of selected recombinant PfEMP1 domains (Table S4) emulsified in AddaVax adjuvant (InvivoGen). Anti-sera were collected one week after the fourth immunization. All animal experiments were approved by the Danish Animal Procedures Committee “Dyreforsøgstilsynet” (permit number 2013-15-2934-00902), and were performed in accordance to the Danish acts LBK 1306 (23/11/2007) and BEK 1273 (12/12/2005).

### Detection of non-immune IgM-binding to recombinant and native PfEMP1 proteins

Non-immune IgM-binding to recombinant PfEMP1 domains was assessed by ELISA as previously described^6^. In brief, flat-bottomed 96-well plates (Thermo Fisher Scientific) were coated overnight (4 °C) with recombinant protein (18 nM) or non-immune IgM (10 nM, for “reverse” ELISA experiments) in Tris saline magnesium buffer. After blocking and washing, plates were incubated (2 h, room temperature) with non-immune human IgM (10 nM) or recombinant protein (18 nM). Bound IgM or protein was detected using HRP-coupled anti-human IgM antibody (1:1,000; DAKO) and anti-His antibody (1:3,000; Qiagen), respectively, followed by TMB PLUS2 ready-to-use substrate (Kem-En-Tec Diagnostics) following the manufacturer’s instructions. Absorbance was read at 450 nm after stopping the colorimetric reaction by the addition of 0.2 M H_2_SO_4_. Binding was considered positive if the measured absorbance was higher than the average absorbance plus three standard deviations for the DBLγ13 (PFD1235w) expressed in an identical way as the constructs assessed in this study. This domain was chosen as a negative control, because erythrocytes infected by parasites expressing that particular variant do not bind non-immune IgM (our unpublished data).

Non-immune IgM-binding to recombinant PfEMP1 domains from *P. falciparum* 3D7 was also assessed by recombinant protein micro-array as previously described^26,27^. In brief, single- or multi-domain proteins were expressed in COS7 cells, purified and immobilized on BioPlex beads as described before^25^. In each experiment, a mixture of 53–55 bead regions with immobilized constructs was used. Beads with immobilized HisAdEx construct^25,59^ were used as negative control. The control contained all the same parts as recombinant domain constructs but short irrelevant 37-mer peptides instead of PfEMP1 domains. Some of the constructs, which showed consistent binding in these high throughput experiments (n = 4–7), were tested then in smaller sets of beads to confirm binding. PCR primers for constructs and protein domain boundaries are shown in Table S6. Uniform coating of the beads was verified using biotinylated anti-GFP antibody (Abcam), followed by incubation with streptavidin-PE (1:250; Jackson Immunoresearch). Non-immune IgM (10 nM; Sigma) binding to bead-bound PfEMP1 constructs (1,000 to 2,000 beads in the reaction) was measured in duplicates on BioPlex 200 machine (BioRad) as described before^25,27^. Binding of non-immune IgM to the constructs were determined by incubation with PE-conjugated anti-human IgM (1:250; Jackson Immunoresearch). The fluorescence signal (MFI, median fluorescence intensity) measured for each control construct was subtracted from the signal of each bead-bound construct, in each of duplicate well, and averaged. Experiments to test IgM binding to bead-immobilized domains were repeated at least three times with qualitatively similar results. Results of representative experiments are shown.

Non-immune IgM binding to native PfEMP1 expressed on the IE surface was detected by flow cytometry as previously described^43^. Briefly, late-stage IEs were purified by MACS and labeled with non-immune human IgM (10 nM; Sigma). IE-bound IgM was measured with a FITC-conjugated anti-human IgM (1:150; Sigma) and ethidium bromide (2 µg/mL) using a Beckman Coulter FC500 flow cytometer for data acquisition. Analysis was performed using FlowLogic software (Inivai Technologies, Australia).

### Selection of infected erythrocytes for surface expression of specific PfEMP1 variants

Antigen-specific IgG was purified from rat sera after immunization with recombinant PfEMP1 domains using HiTrap Protein G HP and NHS-activated HP (coupled to the antigen used for immunization) columns (GE Healthcare) following manufacturer’s instructions. IEs were selected for surface expression of particular IgM-binding PfEMP1 candidates by repeated immune-magnetic selection using PfEMP1-specific rat anti-sera or affinity-purified rat IgG coupled to protein G-Dynabeads (Invitrogen) as described before^60^. Monospecific PfEMP1 expression on IEs was verified by flow cytometry as previously described^43^. In brief, late-stage IEs were labeled with affinity-purified IgG (10 μg/mL) followed by FITC-conjugated goat anti-rat IgG (1:150; Vector Laboratories). Parasite nuclei were stained with ethidium bromide (2 µg/mL) and antibody labeling of IEs quantified by flow cytometry as above.

### Statistical analysis

Data were analyzed by using GraphPad Prism version 8.0.1 (GraphPad Software, San Diego, CA, USA). Two-tailed one-sample t-test relative to construct control was used to analyze the Bioplex results. Spearman’s rank correlation (r_s_) was used to assess the correlation between non-immune IgM-binding and labeling by PfEMP1-specific IgG. P-values < 0.05 were considered significant.

## Supplementary information

Supplementary data
