# Supplementary material for: Comprehensive analysis of Fc-mediated IgM binding to the Plasmodium falciparum erythrocyte membrane protein 1 family in three parasite clones
Source: Sci Rep. 2019 Apr 15;9:6050. doi: 10.1038/s41598-019-42585-0 (PMC6465264; doi:10.1038/s41598-019-42585-0)
Supplement: Supplementary file 1 — Supplementary data [file 41598_2019_42585_MOESM1_ESM.pdf]

Supplementary data for

Comprehensive analysis of Fc-mediated IgM binding to the *Plasmodium falciparum* erythrocyte membrane protein 1 family in three parasite clones

Maria del Pilar Quintana[1], Gertrude Ecklu-Mensah[1,3], Sergey O. Tcherniuk[4], Sisse Bolm Ditlev[1], Andrew V Oleinikov[4], Lars Hviid[1,2#] and Mary Lopez-Perez[1]

Figure S1. Erasure of epigenetic memory by pVBH transfection

Relative proportions of *var* gene transcripts in *P. falciparum* 3D7 (top), HB3 (center), and IT4 (bottom) before pVBH transfection (left), after transfection under blasticidin drug pressure (center), and two weeks after release from drug pressure (right). * As detailed in ref. 1.

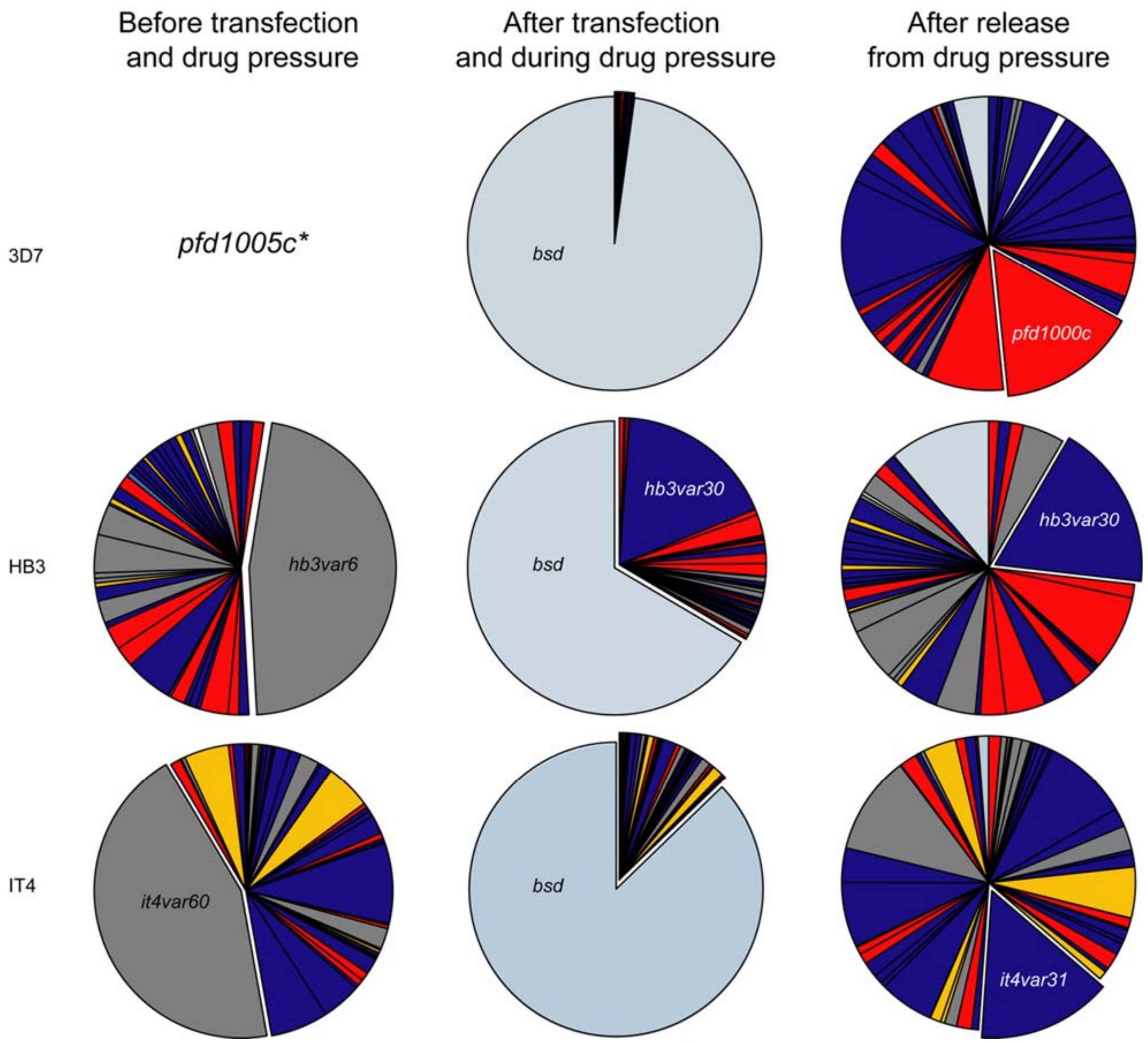

Figure S2. Transcription of* var *genes in selected* P. falciparum *3D7, HB3 and IT4 sub-clones

Data presented as in Fig. 2.

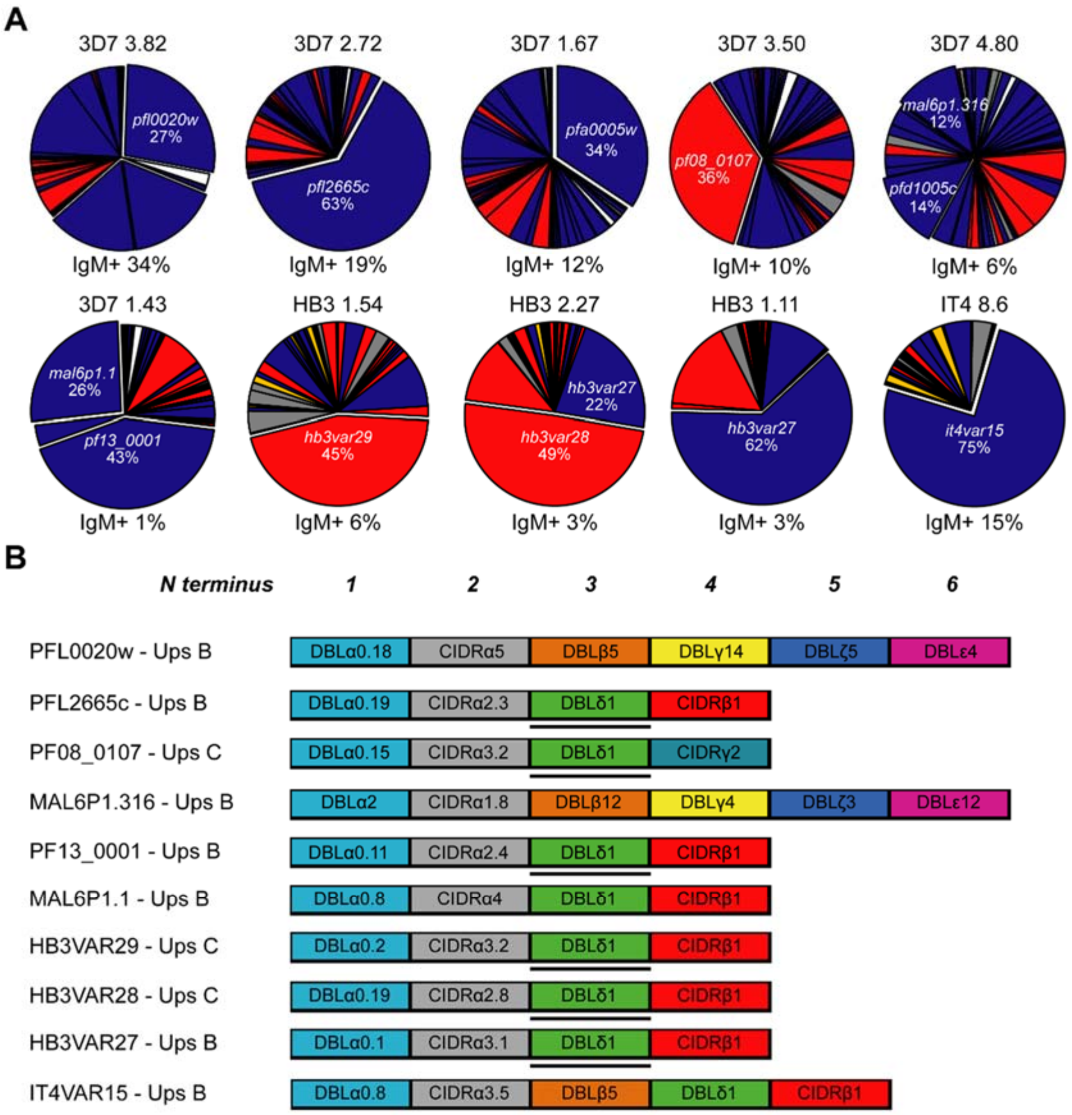

Figure S3. PfEMP1 domains binding to non-immune IgM on ELISA

Data presented as in Fig. 3.

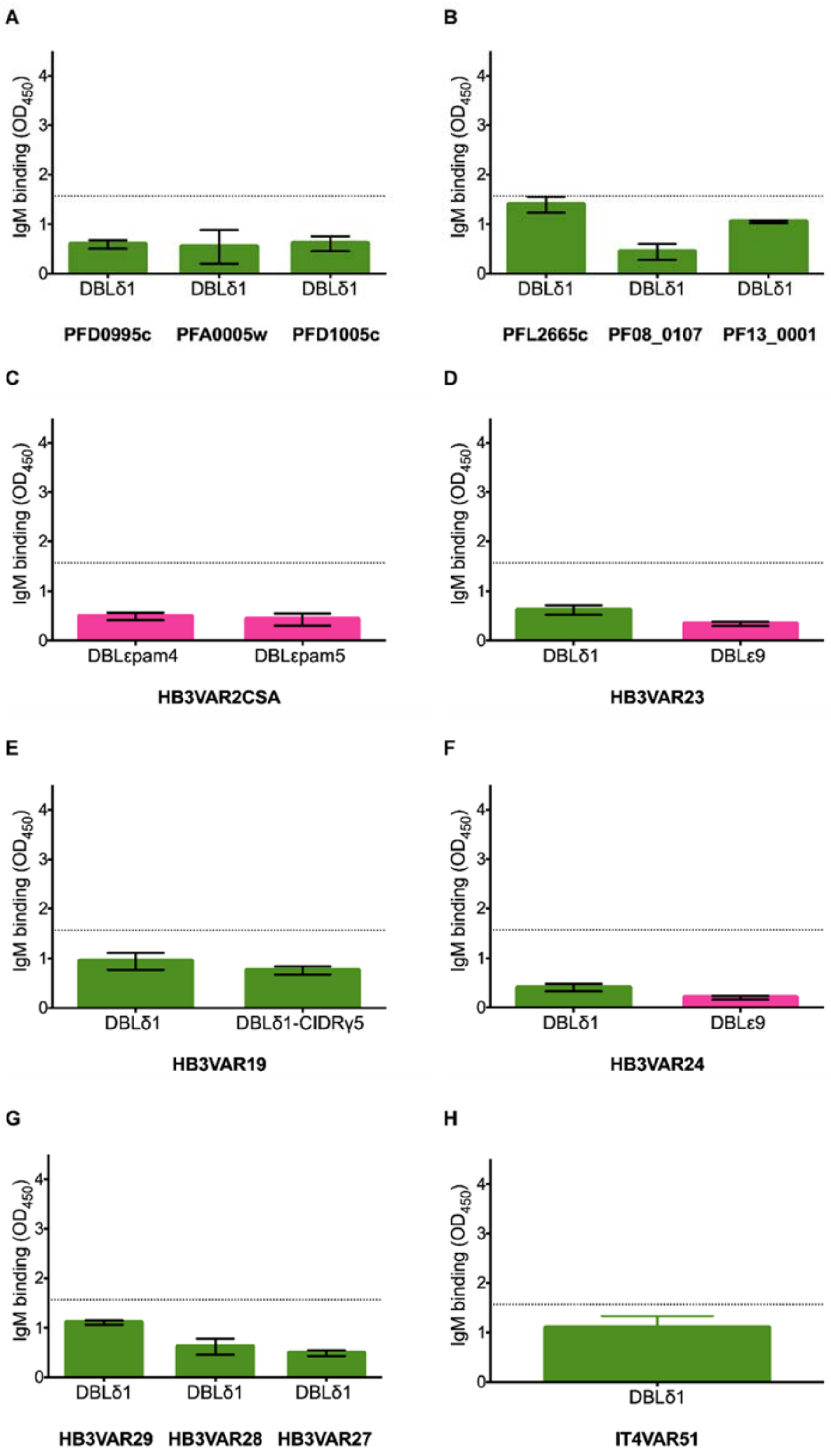

Figure S4. PfEMP1 domains binding to non-immune IgM in a "reverse" ELISA (IgM-coated plates)

Binding of recombinant PfEMP1 domains to immobilized non-immune IgM. Means and standard deviations of data from three independent experiments are shown. Dotted lines represent the cut-off above which binding to non-immune IgM was considered as positive.

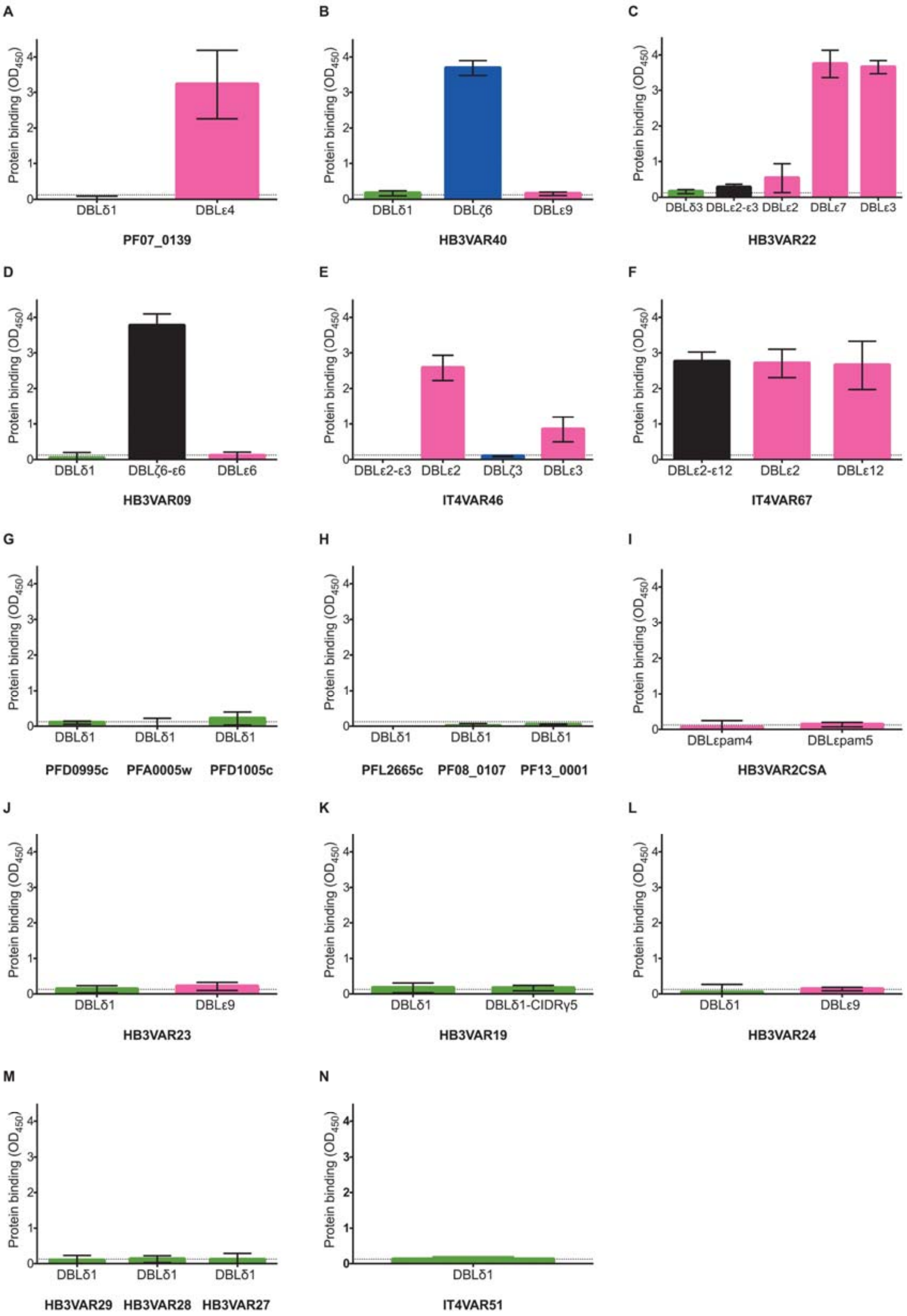

Figure S5. Non-immune IgM-binding to recombinant PfEMP1 domains on BioPlex beads

Non-immune IgM-binding to recombinant domains from PfEMP1 proteins previously (**A**) or not previously (**B**) shown to bind IgM. Data shown are the mean fluorescence intensity (MFI) for each recombinant PfEMP1 construct minus the MFI of the control construct measured in the same well. P-value calculated by t-test relative to control construct. Each experiment was repeated at least three times with quantitatively similar results. Mean and standard deviations of representative experiments are shown. Color-coding as in Fig. 2B. * P< 0.05, ** P< 0.01, *** P< 0.001. The domain structures of the PfEMP1 variants involved are also shown (**C**).

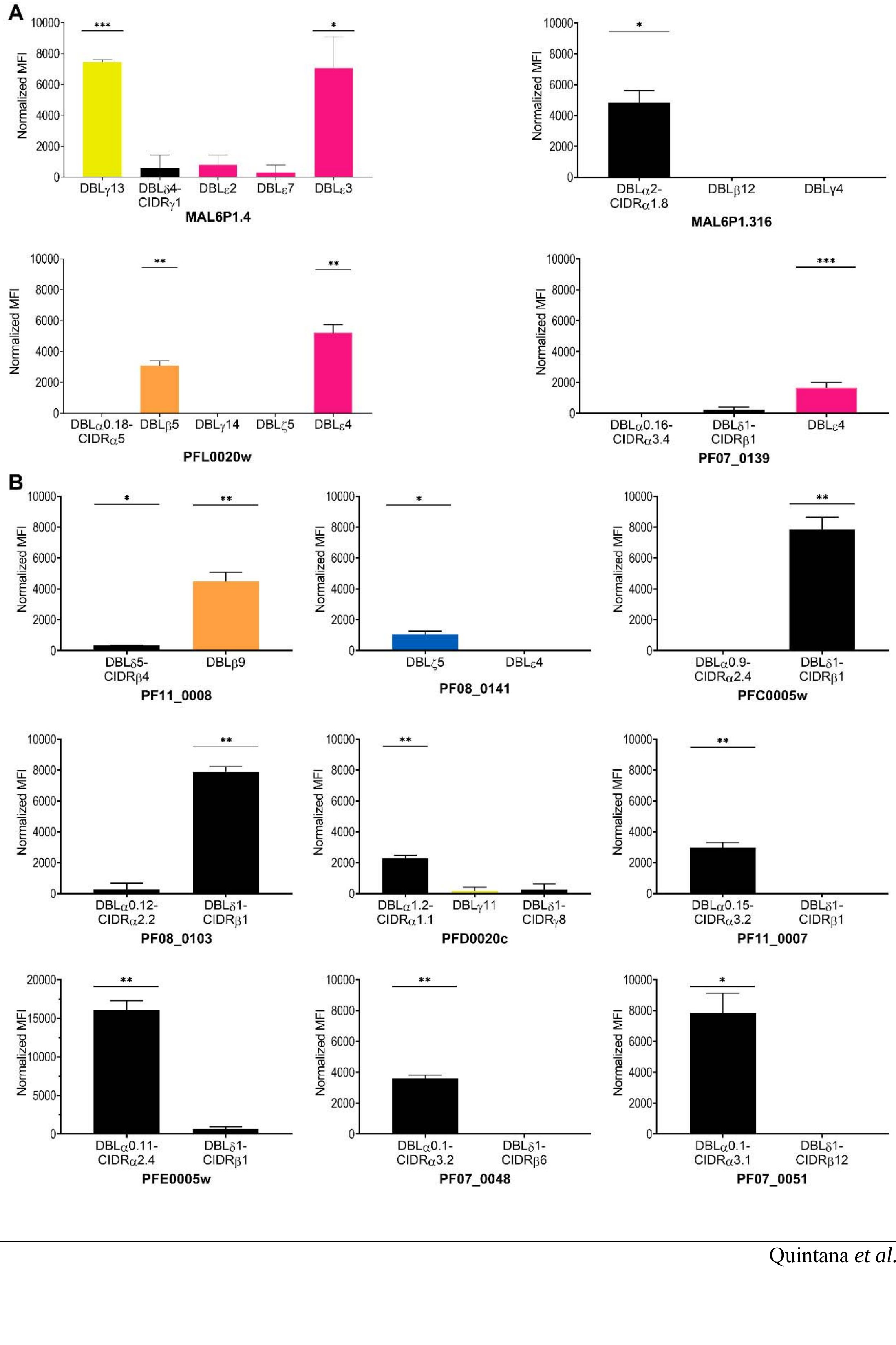

C

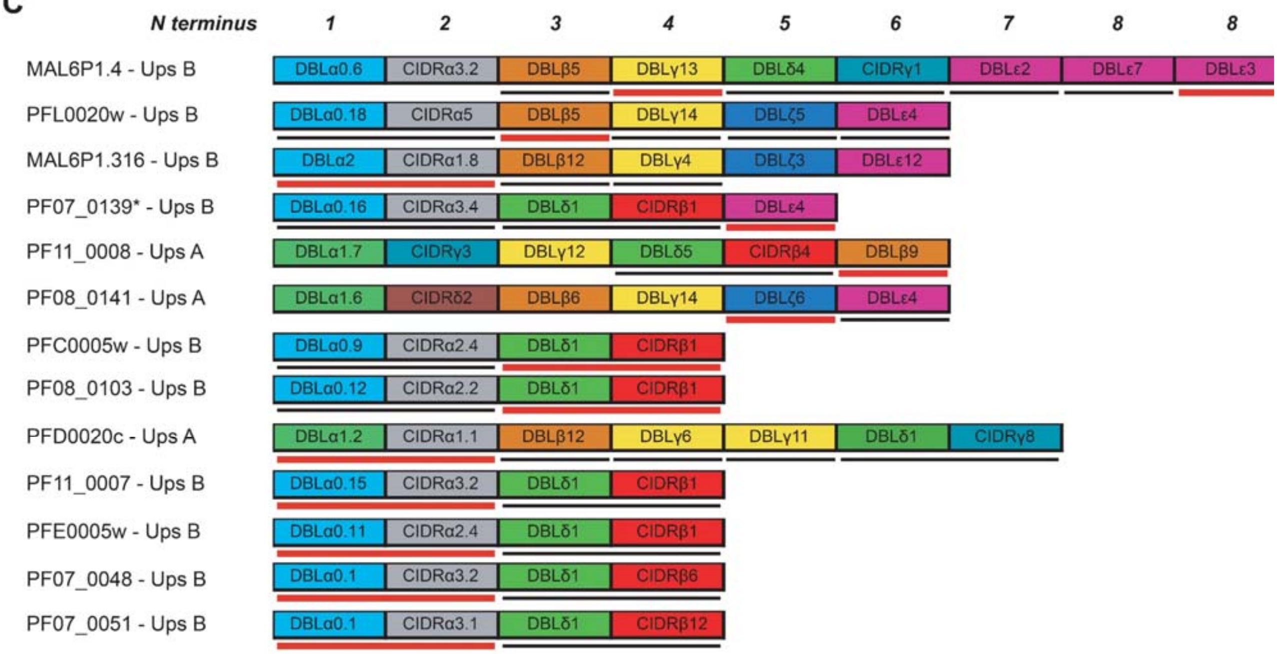
N terminus
1
2
3
4
5
6
7
8
8
MAL6P1.4 - Ups B
DBLα0.6
CIDRα3.2
DBLβ5
DBLγ13
DBLδ4
CIDRγ1
DBLε2
DBLε7
DBLε3
PFL0020w - Ups B
DBLα0.18
CIDRα5
DBLβ5
DBLγ14
DBLζ5
DBLε4
MAL6P1.316 - Ups B
DBLα2
CIDRα1.8
DBLβ12
DBLγ4
DBLζ3
DBLε12
PF07_0139* - Ups B
DBLα0.16
CIDRα3.4
DBLδ1
CIDRβ1
DBLε4
PF11_0008 - Ups A
DBLα1.7
CIDRγ3
DBLγ12
DBLδ5
CIDRβ4
DBLβ9
PF08_0141 - Ups A
DBLα1.6
CIDRδ2
DBLβ6
DBLγ14
DBLζ6
DBLε4
PFC0005w - Ups B
DBLα0.9
CIDRα2.4
DBLδ1
CIDRβ1
PF08_0103 - Ups B
DBLα0.12
CIDRα2.2
DBLδ1
CIDRβ1
PFD0020c - Ups A
DBLα1.2
CIDRα1.1
DBLβ12
DBLγ6
DBLγ11
DBLδ1
CIDRγ8
PF11_0007 - Ups B
DBLα0.15
CIDRα3.2
DBLδ1
CIDRβ1
PFE0005w - Ups B
DBLα0.11
CIDRα2.4
DBLδ1
CIDRβ1
PF07_0048 - Ups B
DBLα0.1
CIDRα3.2
DBLδ1
CIDRβ6
PF07_0051 - Ups B
DBLα0.1
CIDRα3.1
DBLδ1
CIDRβ12

Figure S6. Non-immune IgM-binding and specific PfEMP1 expression on the surface of erythrocytes infected by* P. falciparum *sub-clones

Data presented as in Fig. 5.

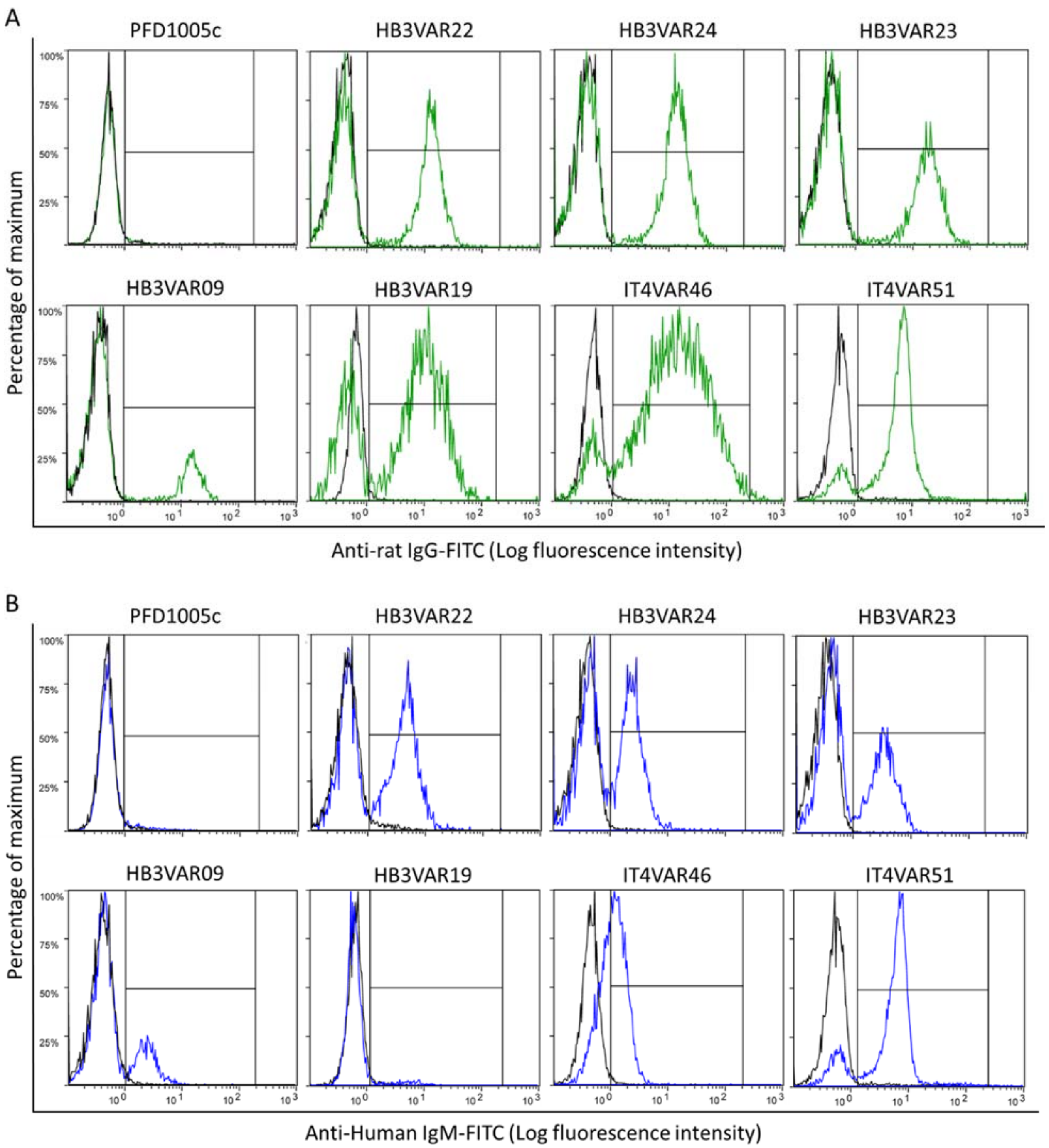

Fig. S7. Non-immune IgM-binding and specific PfEMP1 expression on the surface of infected erythrocytes selected by rat anti-PfEMP1 domain-specific IgG

Data presented as in Fig. 6.

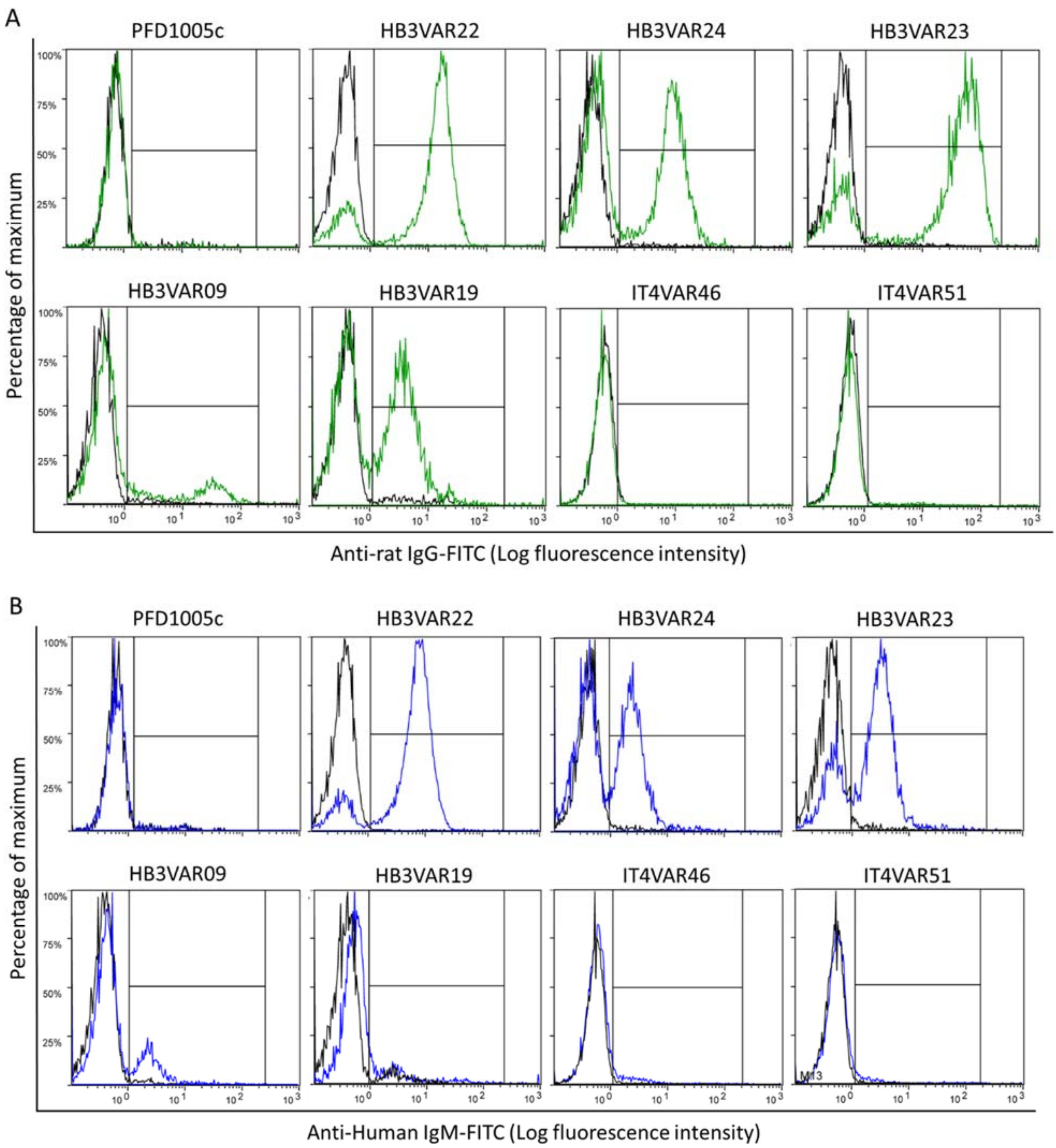

Figure S8. Recombinant protein expression and purification

Coomassie-stained gels (top) and corresponding anti-His immunoblots (bottom) of recombinant proteins from *P. falciparum* 3D7 **(A)**, IT4 **(B)**, and HB3 **(C)**. Arrows indicate the expected size for each construct under reducing conditions (+DTT).

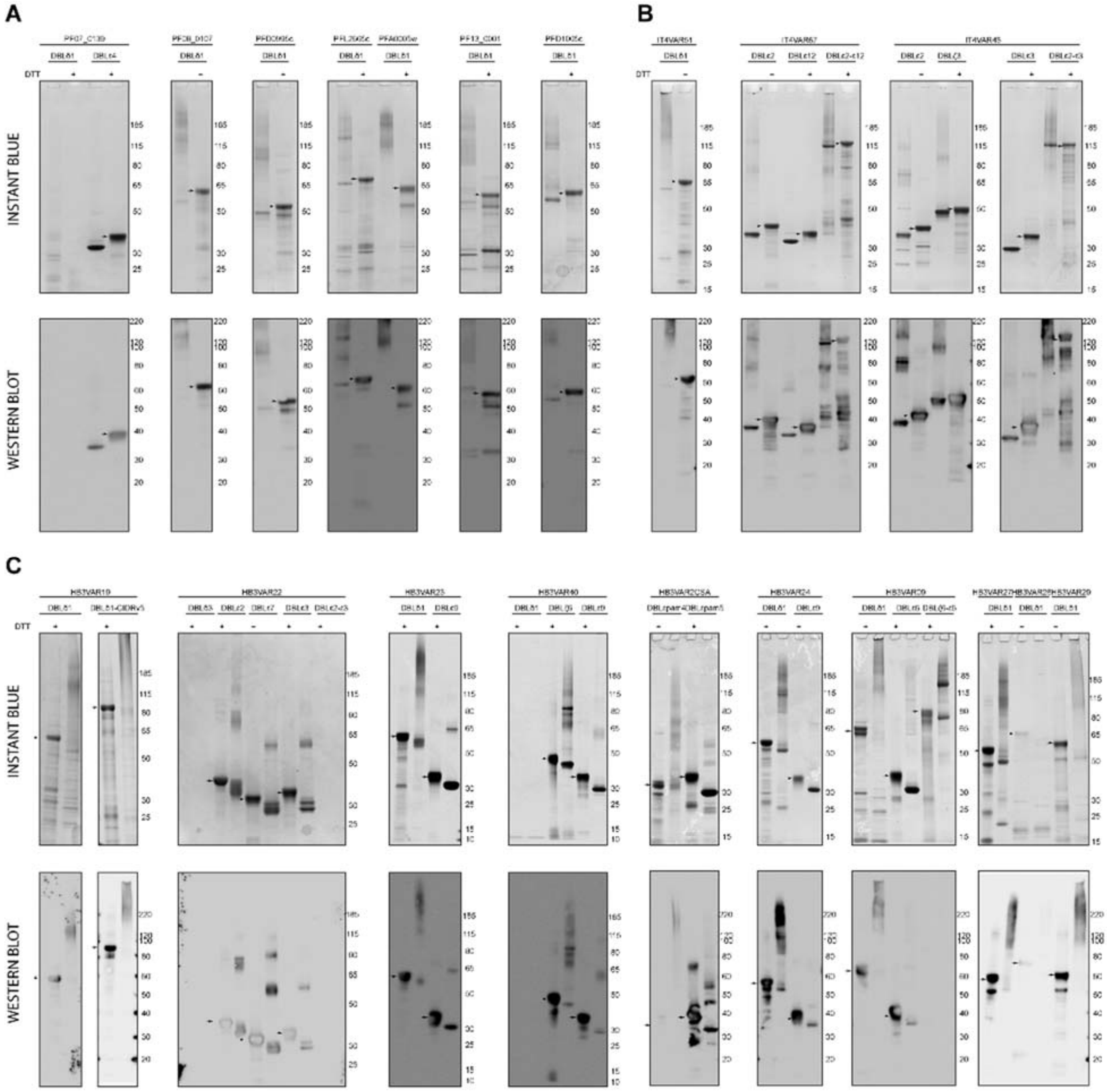

Table S1. **var *gene transcription in 3D7 sub-clones, sorted according to IE reactivity with non-immune IgM***

IgM^{+} IEs	Sub-clone name	Dominant transcripts					
		Primary (%)		Secondary (%)		Tertiary (%)	
79%	**3D7 2.28**	***pfl0030c***	**(86)**	*pfd1000c*	(2)	*pfd1005c*	(2)
63%	**3D7 3.17**	***pfl0030c***	**(61)**	*pfd1005c*	(6)	*pfd1000c*	(4)
34%	3D7 3.82	*pfl0020w*	(27)	*pfl1955w*	(16)	*pfl2665c*	(16)
25%	3D7 3.30	*pfd0995c*	(15)	*pfl0005w*	(9)	*pfd1005c*	(9)
24%	3D7 4.63	*pfd1005c*	(14)	*pfd1000c*	(9)	*pfd1015c*	(7)
23%	3D7 4.34	*pfd1005c*	(14)	*pfd1000c*	(10)	*mal6p1.316*	(5)
19%	**3D7 2.72**	***pfl2665c***	**(63)**	*pfd1005c*	(4)	*pfd1000c*	(4)
19%	3D7 3.75	*pfd1000c*	(30)	*pfd1005c*	(22)	*pfl0030c*	(5)
13%	3D7 2.27	*pfd1000c*	(18)	*pfd1005c*	(13)	*pfd1015c*	(8)
12%	3D7 1.67	*pfa0005w*	(34)	*mal6p1.316*	(8)	*pfd1005c*	(7)
10%	**3D7 3.50**	***pf08_0107***	**(36)**	*pfd1005c*	(8)	*pfd0005w*	(4)
8%	3D7 4.59	*pfd1005c*	(19)	*pfd1000c*	(11)	*mal6p1.316*	(5)
8%	3D7 1.14	*pfl0030c*	(13)	*pfd1005c*	(11)	*pfd1000c*	(10)
7%	3D7 2.68	*pfd1005c*	(13)	*pfd1000c*	(10)	*pfe0005w*	(5)
6%	3D7 4.58	*pfd1005c*	(22)	*pfd1000c*	(8)	*pf08_0107*	(7)
6%	3D7 4.26	*pfd1015c*	(16)	*pfd1005c*	(10)	*pfl0020w*	(10)
6%	3D7 4.80	*pfd1005c*	(14)	*mal6p1.316*	(12)	*pfd1000c*	(6)
5%	3D7 3.11	*pfd1000c*	(33)	*pfd1005c*	(23)	*pfd0625c*	(4)
5%	3D7 3.29	*pfd1005c*	(14)	*pfd1000c*	(9)	*pfd0625c*	(6)
1%	**3D7 1.43**	***pf13_0001***	**(43)**	*mal6p1.1*	(26)	*pfd0625c*	(8)
1%	3D7 3.28	*pfd1005c*	(14)	*pfd1000c*	(9)	*pfd0625c*	(6)
1%	3D7 2.31	*pfd0625c*	(9)	*pfd1005c*	(9)	*pfd1000c*	(9)

Sub-clones with dominant (≥35%) transcript are indicated in boldface. Selected sub-clones presented in Fig. 2 and S2 are indicated by shading.

Table S2.* var *gene transcription in HB3 sub-clones, sorted according to IE reactivity with non-immune IgM

IgM+ IEs	Sub-clone name	Dominant transcripts					
		Primary (%)		Secondary (%)		Tertiary (%)	
72%	**HB3 2.83**	***hb3var2csaA***	**(87)**	*hb3var1csa*	(5)	*hb3var27*	(3)
64%	**HB3 1.43**	***hb3var39***	**(35)**	*hb3var40*	(23)	*hb3var27*	(8)
60%	**HB3 1.26**	***hb3var40***	**(75)**	*hb3var27*	(7)	*hb3var29*	(3)
59%	**HB3 1.83**	***hb3var22***	**(55)**	*hb3var27*	(14)	*hb3var29*	(4)
58%	**HB3 2.6**	***hb3var23***	**(51)**	*hb3var27*	(23)	*hb3var26*	(3)
55%	**HB3 1.12**	***hb3var19***	**(58)**	*Unknown*	(18)	*hb3var27*	(8)
54%	HB3 2.11	*hb3var39*	(22)	*hb3var27*	(18)	*hb3var40*	(17)
54%	HB3 2.41	*hb3var39*	(20)	*hb3var40*	(18)	*hb3var27*	(17)
53%	HB3 1.61	*hb3var39*	(31)	*hb3var40*	(17)	*hb3var27*	(13)
49%	HB3 2.17	*hb3var39*	(12)	*hb3var27*	(9)	*hb3var09/hb3var47*	(8)
49%	HB3 2.68	*hb3var39*	(22)	*hb3var27*	(17)	*hb3var40*	(13)
48%	**HB3 1.16**	***hb3var27***	**(38)**	*hb3var23*	(17)	*hb3var25*	(9)
46%	**HB3 2.94**	***hb3var40***	**(45)**	*hb3var27*	(17)	*hb3var29*	(7)
43%	HB3 1.75	*hb3var39*	(20)	*hb3var40*	(16)	*hb3var10*	(12)
42%	HB3 1.71	*hb3var27*	(24)	*hb3var39*	(16)	*hb3var29*	(9)
41%	HB3 2.42	*hb3var39*	(19)	*hb3var24*	(13)	*hb3var27*	(13)
40%	HB3 1.74	*hb3var27*	(30)	*hb3var39*	(23)	*hb3var34*	(6)
39%	**HB3 2.62**	***hb3var27***	**(38)**	*hb3var39*	(22)	*hb3var40*	(8)
38%	HB3 2.19	*hb3var39*	(23)	*hb3var27*	(20)	*hb3var29*	(11)
37%	HB3 1.40	*hb3var27*	(24)	*hb3var40*	(22)	*hb3var22*	(15)
36%	HB3 2.5	*hb3var27*	(29)	*hb3var29*	(19)	*hb3var22*	(14)
33%	HB3 2.18	*hb3var27*	(27)	*hb3var07*	(15)	*hb3var23*	(13)
27%	**HB3 1.37**	***hb3var27***	**(36)**	*hb3var23*	(28)	*hb3var29*	(11)
26%	HB3 2.26	*hb3var27*	(27)	*hb3var39*	(17)	*hb3var40*	(9)
24%	**HB3 1.25**	***hb3var27***	**(42)**	*hb3var39*	(9)	*hb3var40*	(6)
22%	**HB3 1.84**	***hb3var24***	**(54)**	*hb3var27*	(18)	*hb3var25*	(4)
22%	**HB3 2.71**	***hb3var27***	**(55)**	*hb3var09/hb3var47*	(18)	*hb3var29*	(9)
14%	HB3 1.24	*hb3var39*	(25)	*hb3var40*	(17)	*hb3var29*	(16)
6%	**HB3 1.54**	***hb3var29***	**(45)**	*hb3var27*	(9)	*hb3var08*	(5)
3%	**HB3 1.11**	***hb3var27***	**(62)**	*hb3var29*	(16)	*hb3var24*	(11)
3%	**HB3 2.27**	***hb3var28***	**(49)**	*hb3var27*	(22)	*hb3var29*	(12)

Sub-clones with dominant (≥35%) transcript are indicated in boldface. Selected sub-clones presented in Fig. 2 and S2 are indicated by shading.

Table S3.* var *gene transcription in IT4 sub-clones, sorted according to IE reactivity with non-immune IgM

IgM$^+$ IEs	Sub-clone name	Dominant transcripts					
		Primary (%)		Secondary (%)		Tertiary (%)	
82%	**IT4 7.3**	***it4var46***	**(62)**	*it4var51*	(27)	*it4var65*	(2)
80%	**IT4 8.51**	***it4var51***	**(70)**	*it4var21*	(8)	*it4var59*	(6)
79%	**IT4 7.37**	***it4var51***	**(67)**	*it4var34*	(13)	*it4var59*	(4)
78%	**IT4 7.72**	***it4var46***	**(86)**	*it4var21*	(2)	*it4var59/65*	(2)
67%	**IT4 8.1**	***it4var67***	**(76)**	*it4var21*	(6)	*it4var59*	(3)
63%	**IT4 8.7**	***it4var67***	**(57)**	*it4var51*	(19)	*it4var46*	(5)
60%	**IT4 8.3**	***it4var67***	**(50)**	*it4var34*	(19)	*it4var66*	(15)
59%	**IT4 7.5**	***it4var67***	**(81)**	*it4var59*	(7)	*it4var21*	(7)
50%	**IT4 7.4**	***it4var67***	**(60)**	*it4var51*	(14)	*it4var65*	(5)
39%	**IT4 8.5**	***it4var51***	**(84)**	*it4var59*	(2)	*it4var21*	(2)
39%	**IT4 8.34**	***it4var46***	**(91)**	*it4var34*	(1)	*it4var21*	(1)
37%	IT4 7.38	*it4var21*	(27)	*it4var59*	(23)	*it4var65*	(20)
15%	**IT4 8.6**	***it4var15***	**(75)**	*it4var67*	(5)	*it4var08*	(4)

Sub-clones with dominant (≥35%) transcript are indicated in boldface. Selected sub-clones presented in Fig. 2 and S2 are indicated by shading.

Table S4. Recombinant proteins used for rat immunization.

PfEMP1 variant	Domain name	Non-immune IgM binding by ELISA
HB3VAR09	DBLε6	-
HB3VAR19	DBLδ1-CIDRγ5	-
HB3VAR22	DBLε7	+
	DBLε3	+
HB3VAR23	DBLε9	-
HB3VAR24	DBLε9	-
HB3VAR40	DBLζ6	+
PFD1005c	DBLδ1	-
PF07_0139	DBLε4	+
IT4VAR46	DBLε3	+
IT4VAR51	DBLδ1	-
IT4VAR67	DBLε2	+

Table S5. List of primers used to clone various PfEMP1 domains for recombinant protein expression

	Construct Name*	Cloning Primers	Domain size (aa)
1	PFD0995c DBLδ1	AT**GGATCC**TGCAATATAGTAAAAACACTATTTGAGAGC<br>CTA**GCTAGCTTA**ACATGTTTTACAATACGTTGAAAGAGAA	446
2	PFA0005w DBLδ1	CTA**GCTAGC**TGCAATATAGTGAACAATGTGTTTACAG<br>CTA**CTCGAGTTA**ACATGGAGCACAATAATTTTCATGT	508
3	PFD1005c DBLδ1	AT**GGATCC**TGCAATATAGTAAAAACACTATTTGAGAGC<br>CTA**GCTAGCTTA**ACATGTTTTACAATACGTTGAACGAG	496
4	PFL2665c DBLδ1	AT**GGATCC**TGCGAAACAGTGGCCACAG<br>CTA**GCTAGCTTA**ACATGGTTTACAATTATCTGCATGTTT	563
5	PF08_0107 DBLδ1	AT**GGATCC**TGTGAAATAGTACAAAAACTATTTACTAATGG<br>CTA**GCTAGCTTA**ACACGCTTTACAATACGTTGAACG	519
6	PF13_0001 DBLδ1	CTA**GCTAGC**TGCAAAACGGTGGCCG<br>CTA**CTCGAGTTA**ACATGGATCACAATATTTTGCGT	482
7	PF07_0139 DBLδ1	GC**GGATCC**TGCAAAACGGTGGCAGAA<br>CTA**GCTAGCTTA**ACATGGATCACAATATTTTTATGTCC	487
8	PF07_0139 DBL1ε4	GC**GGATCC**ATATTTAAAACATGTCCTTATGATAATGATAC<br>CTA**GCTAGCTTA**GCACTCACATTTACTTTTCAGATTTTTA	322
9	HB3VAR2CSA DBLεpam_D4	AT**GGATCC**GAACAAGTAAAATATTACAAATATAATAATGCTG<br>CT**AGCTAGCTTA**TTCCGATTCTTTGTTACTTTTACGG	312
10	HB3VAR2CSA DBLεpam_D5	AT**GGATCC**CCATTAGATAGATGCTTTGACGACA<br>CT**AGCTAGCTTA**ACACGGACATTTAGAACAATGTTCC	302
11	HB3VAR40 DBLδ1	AT**GGATCC**TGTGAAATAGTTGACGAGCTATTTAAAG<br>CTA**GCTAGCTTA**ACACGCTTTACAATACGTTGAAGG	475
12	HB3VAR40 DBLζ6	CTA**GCTAGC**TGTGTTGAAAGAGCGGCAAAAC<br>CTA**CTCGAGTTA**ACATTCACATGCTCTTTCATATTCTTT	417
13	HB3VAR40 DBLε9	AT**GGATCC**CCATTGGATCAATGTCCTCATACT<br>CTA**CTCGAGTTA**ACATTCACATTTTTCTTTATTTTTAGTATCG	321
14	HB3VAR22 DBLδ3	AT**GGATCC**TGCAACACAGTGAAAACCGC<br>CTA**GCTAGCTTA**ACATGTTTTGCAATATTCTGAAGAAC	465

15	HB3VAR22 DBLε2	AT**GGATCC**CCGAAAATATTGTGGTATTACTAAAAC<br>CTA**GCTAGCTTA**ACATTCACATTCATCTTTAACATCATCA	348
16	HB3VAR22 DBLε7	AT**GGATCC**GACCTAGATAAATGTCCAACTGAAGAA<br>CTA**GCTAGCTTA**ACATTGACATTCAGAACATTTTTCTTT	275
17	HB3VAR22 DBLε3	AT**GGATCC**CCTTATCTTCGTTGTCCTGATTCAAAATTATGT<br>CTA**GCTAGCTTA**GCAATCACATTTATCTTTATATGTAGGCT	303
18	HB3VAR22 DBLε2-DBLε3	AT**GGATCC**CCGAAAATATTGTGGTATTACTAAAAC<br>CTA**GCTAGCTTA**GCAATCACATTTATCTTTATATGTAGGCT	979
19	HB3VAR23 DBLδ1	AT**GGATCC**TGTGACATAGTAAACACACTATTTAGTGACAC<br>CTA**GCTAGCTTA**ACATGCTTTACAATACGTTGAAGGAG	476
20	HB3VAR23 DBLε9	AT**GGATCC**CCATTGGATAAATGCCCTAATACT<br>CTA**GCTAGCTTA**ACAGTTACATTTATTTTTCAGGTTGTTATC	321
21	HB3VAR19 DBLδ1	AT**GGATCC**TGCAGTATAGTGGCTGACATATTTAAA<br>CTA**GCTAGCTTA**ACAGGTTTCGCAATATTTTGAATG	494
22	HB3VAR19 DBLδ1-CIDRγ5	AT**GGATCC**TGCAGTATAGTGGCTGACATATTTAAA<br>CTA**GCTAGCTTA**ACACGTTTGTGCTTGGTTATCG	769
23	HB3VAR24 DBLδ1	AT**GGATCC**TGCGACACAGTGAAAAGCGC<br>CTA**GCTAGCTTA**ACACGTTTTACAATATTCTAAAGGACCA	453
24	HB3VAR24 DBLε9	AT**GGATCC**CCATTGGATCAATGTCCTCATACA<br>CTA**GCTAGCTTA**ACAGTTACATTTATTTTTCAGGTTGTTATC	322
25	HB3VAR09 DBLδ1	AT**GGATCC**TGTGACATAGTAAAAGAACTATTTAAAGACAC<br>CTA**GCTAGCTTA**ACACGTTTTACAATAATCTAAGGGGC	531
26	HB3VAR09 DBLζ6	CTA**GCTAGC**TGTGTTGAGAGAATAGCAAAAGAATTAA<br>CTA**CTCGAGTTA**ACATTCACATACTTTTTCATATTCTTTTG	424
27	HB3VAR09 DBLε6	CTA**GCTAGC**CCATTAGATAATTGTCCTAAGGATAATAAAC<br>CTA**CTCGAGTTA**ACAAACACATATATTTTTAACATCACTATCG	319
28	HB3VAR29 DBLδ1	AT**GGATCC**TGCGAAACAGTGAAAAAAGCAC<br>CTA**GCTAGCTTA**ACATGGATCACAAAGATTTGTATGTTT	490
29	HB3VAR28 DBLδ1	AT**GGATCC**TGCCCAATAGTAAAAACCGCACTT<br>CTA**GCTAGCTTA**ACATGGATCACAATGTTTTGTATGTTT	539
30	HB3VAR27 DBLδ1	GC**GGATCC**TGTGAAATAGTACAAACACTATTTACTAGTGG	473

		CTA**GCTAGCTTA**ACATGGATTACAATCTTTTGCATGT	
31	IT4VAR46 DBLε2	GC**GCTAGC**TCGGATATATTTTGTGATCGTAACAA<br>CTA**CTCGAGTTA**ACACGTACACAAATTTTTGACTTCAT	347
32	IT4VAR46 DBLζ3	GC**GCTAGC**TGTATAGAGAAAGCTGCATATGAACTACA<br>CTA**CTCGAGTTA**ACACTTGCATTTTTCTTTGTATACTTTAG	431
33	IT4VAR46 DBLε3	GC**GCTAGC**CCATTAGATAAATGTCCTGATCAAAATAC<br>CTA**CTCGAGTTA**GCAATTACATTTACCTTTAAGTGCTTTAT	309
34	IT4VAR46 DBLε2-DBLε3	GC**GCTAGC**TCGGATATATTTTGTGATCGTAACAA<br>CTA**CTCGAGTTA**GCAATTACATTTACCTTTAAGTGCTTTAT	1104
35	IT4VAR51 DBLδ1	GC**GCTAGC**TGTGACATAGTGGCAACACTATTTGA<br>CTA**CTCGAGTTA**ACACGTTTTACAATACGTTGAACGA	588
36	IT4VAR67 DBLε2	AT**GGATCC**TCGGATAGGTTGTGTGATGATAAGA<br>CTA**GCTAGCTTA**ACACGTACACAACATTTTGACTTCAT	345
37	IT4VAR67 DBLε12	AT**GGATCC**CCATTAGATGATTGTCCCGACG<br>CTC**GCTAGCTTA**ACAAATATATTTTAATATTTCTTTATATGCGAC	325
38	IT4VAR67 DBLε2-DBLε12	AT**GGATCC**TCGGATAGGTTGTGTGATGATAAGA<br>CTC**GCTAGCTTA**ACAAATATATTTTAATATTTCTTTATATGCGAC	1129

Sequences recognized by the restriction enzymes used for cloning are presented in bold. A stop codon was included in the reverse primer. The enzymes BamHI (GGATCC), NheI (GCTAGC) or XhoI (CTCGAG) were used. *Names, sequences and domain boundaries as reported in the VarDom 1.0 server. (http://www.cbs.dtu.dk/services/VarDom/)

Table S6. PfEMP1 constructs from* P. falciparum *3D7 cloned into pHisAdEx vector*

	PfEMP1	Construct name	Cloning primers	Domain size (aa)
1	PFD0020c	DBLα1.2 -CIDRα1.1 (AA 2-745)	F: CCC**GGATCC**AGGGGACAGGTTCATCAACTCCTTCGG	743
			R: CCC**TCCGGA**GCCACCATTATTGTTTGTTCCACA	
2		DBLβ12 (AA 736-1192)	F: CCC**GGATCC**AGAACCCCTGTGGAACAAACAATAATGG	456
			R: CCC**GAATTC**CTGGTTTTTGTGCCTTCTGCCTAG	
3		DBLγ6 (AA 1211-1536)	F: CCC**GGATCC**AGGGCGGAACAGGTGGAATAGATGG	325
			R: CCC**GAATTC**CCTCGGAGTTATTTTTCACTTTTTCC	
4		DBLγ11 (AA 1583-1901)	F: CCC**GGATCC**AGAGCGCAACTGATTATATAGAAAAG	318
			R: CCC**GAATTC**CCTTATACTCACAATTTGCACTAGTA	
5		DBLδ1-CIDRγ8 (AA 2119-2894)	F: CCC**GGATCC**AGAAATATGGCGGAAACAACTCACGTTT	775
			R: CCC**GAATTC**CTGCTGGGTTTTGGGCACTAAGTA	
6	PF08_0141	DBLζ5 (AA 1700-2077)	F: CCC**GGATCC**AGCCTTCTATTGAAGAATGTGATATCAA	377
			R: CCC**GAATTC**CATCCAATGGATCAAAATCTTTCGCAC	
7		DBLε4 (AA 2080-2387)	F: CCC**GGATCC**AGCCTGTTGATAATAACGAATGTAAGAAG	307
			R: CCC**TCTAGA**AGGTGAATCTTTTTTTTTTTCTTTTTCCG	
8	PFL0020w	DBLα0.18 CIDRα5 (AA 2-731)	F: CCCC**CTTAAG**ACATCATGTAGTCCGGAGAATAC	279

			R: CCC**GAATTC**CCTCTTTGGAGCAATTTTTTCTTCTCG	
9		DBLβ5_D4 (AA 750-1242)	F: CCC**GGATCC**AGAATCCGTGTAGCGCCCAACCTGG	492
			R: CCC**GAATTC**CCGCATCTTTTTTTCTTCTTCCTTC	
10		DBLγ14 (AA 1255-1592)	F: CCC**GGATCC**AGGCAACAGATCCTATAGATGGGTG	337
			R: CCC**GAATTC**CTGGTTGTCTCGACGCATTTTCC	
11		DBLζ5 (AA 1698-2041)	F: CCC**GGATCC**AGGCAAATAATTTATTGGACACATCTTG	343
			R: CCC**GAATTC**CATCAGTTTTAACAAATTTAAATCTTCGAC	
12		DBLε4 (AA 2073-2382)	F: CCC**GGATCC**AGCCTGTTGACAAGGACGAATGC	309
			R: CCC**GAATTC**CTCCATTCCCACTTTTATCGTATTTTTC	
13	MAL6P1.4	DBLβ5 (AA 830-1309)	F: CCC**GGATCC**AGAGTAATCCATGTGCTACTCCTAG	479
			R: CCC**TCTAGA**CGGAGGCACTTGTGGCCTAC	
14		DBLγ13 (AA 1328-1660)	F: CCC**GGATCC**AGGGTAAGGATGGTGAAACAACAGAAA	332
			R: CCC**GAATTC**CACTAGAATCTTCATTATGTTGTTGTG	
15		DBLδ4-CIDRγ1 (AA 1710-2388)	F: CCC**GGATCC**AGACTGAAGCTTGTTCAACAAAATATAAAAAT	678
			R: CCC**GAATTC**CAGAGGTTTCTTCTTCGTCACTATG	
16		DBLε2 (AA 2413-2774)	F: CCC**GGATCC**AGCCCAAAACAGATTCCGATATTTTG	361
			R: CCC**GAATTC**CATCATTAACTTTATCGTCATGTGC	
17		DBLε7 (AA 2785-3063)	F: CCC**GGATCC**AGAATAAAAATATATGTAATAAATATAAAAAACGACG	278
			R: CCC**GAATTC**CCTCTATATCTTTAAAACTACATATAC	

18		DBLε3 (AA 3105-3410)	F: CCC**GGATCC**AGCCTGAAGACATTGAATGTAGTCAA<br>R: CCC**TCTAGA**AGGTGTTGTGGGAATCATTTTTTG	305
19	MAL6P1.316	DBLα2-CIDRα1.8 (AA 2-726)#	F: CCCC**TCCGGA**GCGCCGCAACGTGCCGAAGATTC<br>R: CCC**GAATTC**CACCACGAGTGGGTATACGAGTGCG	724
20		DBLβ12 (AA 703-1204)	F: CCC**GGATCC**AGGAAGAAGATGAAAATTGCCCC<br>R: CCC**GAATTC**CACCTCCAGTGGGTTTTGGTGG	501
21		DBLγ4 (AA 1265-1603)	F: CCC**GGATCC**AGTCAAATGGTGGAAAAAATGGAATAAATGG<br>R: CCC**TCTAGA**TGGTTGTCTCGACGCATTTTCCATG	338
22	PF07_0139	DBLα0.16-CIDRα3.4 (AA 2-918)	F: CCC**GGATCC**AGGCGAGGCCATCAGGTAGTGCTGG<br>R: CCC**TCTAGA**GGCGTCAGTAAGACTGGTTTTGAG	916
23		DBLδ1-CIDRβ1 (AA 910-1688)	F: CCC**GGATCC**AGGCACTCAAAACCAGTCTTACTGAC<br>R: CCC**GAATTC**CTTCACTTTCTGGAACTTTCGATTTTTCC	778
24		DBLε4 (AA 1722-2011)	F: CCC**GGATCC**AGTATCGAAATAAAAATAACATTGGATGTCC<br>R: CCC**GAATTC**CTTTATCGTCGTCAATAAAATTTTGAACG	289
25	PF11_0008	DBLδ5-CIDRβ4 (AA 1236-1874)	F: CCC**GGATCC**AGAAATATGGGAAGATGCCACTAGGTTT<br>R: CCC**GAATTC**CATTTTTACAATGACGTATTTCATTTTTAAG	638
26		CIDRβ4 (AA 1922-2420)	F: CCC**GGATCC**AGAATCCATGTGGCGACAAAAGCGCC<br>R: CCC**TCTAGA**TGCCGGCTCCTCCTCCTCTTCGGC	498
27	PFC0005w	DBLα0.9-CIDRα2.4 (AA 2-854)	F: CCC**GGATCC**AGGTTAGGACATTAGATCCTGAGG	852

			R: CCC**TCTAGA**AGCAGTGTTGTCGCCATTAG	
28		DBLδ1-CIDRβ1 (AA 848-1685)	F: CCC**CTTAAG**ACTAATGGCGACAACACTGCTC	837
			R: CCC**TCTAGA**TTCCTCGGATCGACTATCTTCC	
29	PF08_0103	DBLα0.12-CIDRα2.2 (AA 2-815)#	F: CCC**GGATCC**AGTCGACGCTAGGAGGTGGTACTGAC	813
			R: CCC**GAATTC**CGGCGGCATTCAAGTCGCCTTTGG	
30		DBLδ1-CIDRβ1 (AA 810-1616)	F: CCC**CTTAAG**GGCGACTTGAATGCCGCCTGCAC	806
			R: CCC**GAATTC**CTGATGCCGGTGTACAAGTTTCTCC	
31	PF11_0007	DBLα0.15-CIDRα3.2 (AA 2-844)	F: CCC**GGATCC**AGGAGCCGCATGGAGGTAGCGG	842
			R: CCC**GAATTC**CTTCCTCCTCCTCCTCCTGCTCTTC	
32		DBLδ1-CIDRβ1 (AA 908-1700)	F: CCC**GGATCC**AGAAATACGGTCCAAAGGCACCCAC	792
			R: CCC**GAATTC**CGCCTTTTTCCTCTGCTTTTGGTTC	
33	PFE0005w	DBLα0.11-CIDRα2.4 (AA 2-793)	F: CCC**GGATCC**AGGGGCCGCCAGGTATTACTGGTAC	791
			R: CCC**GAATTC**CTTCTTCCTTTTCCTCAGTGGCC	
34		DBLδ1-CIDRβ1 (AA 862-1706)	F: CCC**CTTAAG**GACAATCTGAAAGAAGCCTGCAC	844
			R: CCC**GAATTC**CTCCTTTGTCTTTTTCCTCCTCTTG	
35	PF07_0048	DBLα0.1-CIDRα3.2 (AA 2-901)	F: CCC**GGATCC**AGGCGAGGCCAGGTAGCGGTGGTGG	899
			R: CCC**GAATTC**CTGGGTTCTGAAAGAGTTTAGACAC	
36		DBLδ1-CIDRβ6 (AA 901-1679)	F: CCC**GGATCC**AGCCAAACAATTTTTCCGACGCTTGTA	778

			R: CCC**GGCGCC**TGCTGCTGCTGGTGTACAAGTCTC	
37	PF07_0051	DBLα0.1-CIDRα3.1 (AA 2-901)	F: CCC**GGATCC**AGGCGCCAGGAGGTCGTCAGGGTGAT	899
			R: CCC**GAATTC**CTGCTTGTTCCACGATTTTGCAAACGTC	
38		DBLδ1-CIDRβ1 (AA 910-1714)	F: CCC**GGATCC**AGGCCGCCTGTACCCTCAAATATGG	804
			R: CCC**GAATTC**CGGTTTCCGCACTAGGTGCTGCTG	

*Sequences recognized by the restriction enzymes used for cloning are presented in bold.

Only primers for constructs presented in Fig. S5 are included in the table. Primers for remaining constructs are available upon request to Andrew Oleinikov.

# indicates two exceptions, where construct was cloned into pHisAdEx vector for expression and functional testing.

Reference for Supplementary data

1 Fastman, Y., Noble, R., Recker, M. & Dzikowski, R. Erasing the epigenetic memory and beginning to switch - the onset of antigenic switching of *var* genes in *Plasmodium falciparum*. *PLoS One* **7**, e34168, doi:10.1371/journal.pone.0034168 (2012).
